# Characteristics of Phenotypic Variation of *Malus* Pollen at Infrageneric Scale

**DOI:** 10.3390/plants13172522

**Published:** 2024-09-08

**Authors:** Junjun Fan, Yun Wang, Zhenping Hao, Ye Peng, Jingze Ma, Wangxiang Zhang, Mingming Zhao, Xueming Zai

**Affiliations:** 1 College of Horticulture, Jinling Institute of Technology, Nanjing 210038, Chinazxm1@jit.edu.cn (X.Z.); 2 College of Life Sciences, Nanjing Forestry University, Nanjing 210037, China; pengye@njfu.edu.cn; 3 College of Forestry and Grass, Co-Innovation Center for Sustainable Forestry in Southern China, Nanjing Forestry University, Nanjing 210037, China

**Keywords:** *Malus*, pollen morphology, genetic relationship, family aggregation distribution, variation trend

## Abstract

Pollen carries extensive genetic information, which may provide clues regarding the kinship of *Malus,* whose genetic relationships are complex. In this study, the phenotypic variation of pollen from 107 *Malus* taxa was investigated using combined methods of intraspecific/interspecific uniformity testing, cluster analysis, and Pearson correlation analysis. The family aggregation distributions in *Malus* sections, species, and cultivars were analyzed to infer their pedigree relationships. The results showed that (1) compared with pollen size and morphology, aberrant pollen rate and ornamentation were highly interspecifically differentiated, but ornamentation was also intraspecifically unstable, especially perforation densities ($\bar{c.v.}$ > 15%). (2) The pollen alteration direction from the original to the evolutionary population of *Malus* was large to small, with elliptic to rectangular morphologies, large and compact to small and sparse ridges, and low to high perforation densities. However, there was no significant change in pollen size. (3) The 107 studied taxa were divided into four groups. *Malus* species were relatively clustered in the same section, while homologous cultivars showed evidence of family aggregation distribution characteristics (92.60% of cultivars were clustered with their parents). (4) *M*. *baccata* and *M*. *pumilar* var. *neidzwetzkyana* were high-frequency parents, participating in 38.7% and 20.7% of cross-breeding, respectively. Overall, this study provides a reference for identifying *Malus*’ pedigree relationship.

## 1. Introduction

Pollen morphology has developed during long-term evolution and exhibits species-specific characteristics [1,2]. Therefore, pollen traits are often used in investigations on plant distribution, genetic evolution, or systematic taxonomy [3,4,5]. Previous studies mainly used morphological comparisons, cluster analyses, or principal component analyses of pollen phenotypic parameters [6,7,8] and then deduced evolutionary relationships based on similarity or clustering genetic distance. Alternatively, some researchers mapped morphological structures onto a molecular phylogenetic tree to analyze the evolutionary trends of phenotypes [9,10]. However, most researchers only use descriptive methods to illustrate these results, without statistical analyses to support their conclusions. The systematization of germplasm materials is an essential basis for the statistical analysis of phylogenetic data. Previous studies mainly analyzed the genetic relationships among species, genera, or families [11,12,13,14]. However, there is value in exploring the relationship between parent and progeny and taxonomic sections below the level of the genus or even the species.

*Malus* spp. are of high ornamental value, and there is a rich diversity of *Malus* germplasm globally [15]. More than 30 species have been recorded [16,17], and nearly one thousand *Malus* taxa have been bred in the past 200 years, but only over 200 cultivars can be found in the nurseries [15,18]. Unfortunately, the genetic backgrounds and inter-/intra-species relationships of most cultivars are still unclear [6]. So far, researchers have identified ancestors for 65 cultivars, which provides a systematic basis for the statistical analysis of the phylogenetic relationship of the genus [19,20].

Based on pollen phenotypes of 107 *Malus* taxa (23 species and 84 cultivars), we combined several statistical analysis methods to examine *Malus* plant classification and pedigree relationships at two levels: classification units (sections, species, and cultivars) and parent–progeny populations. The objectives are (1) to clarify the characteristic of phenotypic variation of pollen from the original to the evolutionary *Malus* population; (2) to explore the family aggregation distribution characteristics of *Malus* classification units and parent–progeny populations; and (3) to investigate how the frequency of hybridization with important parents affects the affinity of *Malus* cultivars.

## 2. Results

### 2.1. General Characteristics of Malus Taxa Pollen

**Figure 1:**
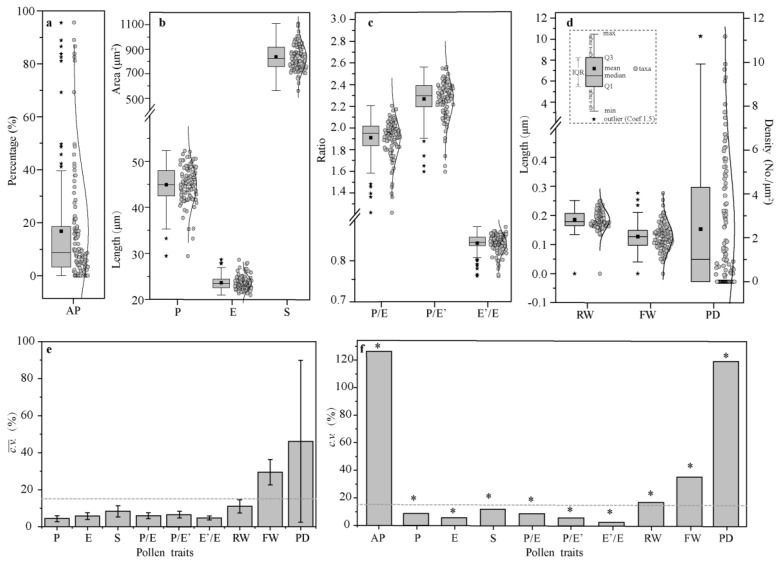
The box plots and coefficient of variation of pollen morphology traits for *Malus* taxa. (**a**) The box plot of the percentage of aberrant pollen grains. (**b**) The box plot of the relative pollen size. Indicators contain the length of the polar axis (P), equatorial diameter (E), and area of equatorial view with two colpi (S). (**c**) In the box plot of pollen morphology, indicators include P/E, P/E′, and E′/E. E′ indicates the diameter at the equatorial plane halfway between the equator and pole. (**d**) The box plot of pollen ornamentation. The indicators measured were the ridge width (RW), furrow width (FW), and perforation density (PD). The interquartile range (IQR) is the box plot (box body) showing the middle 50% of observation values and can be calculated by subtracting the lower quartile (Q1) from the upper quartile (Q3). An outlier (★) is an observation value numerically distant from the rest of the data, 1.5 times the interquartile range, less than Q1 and greater than Q3. The min (—) and max (—) values (excluding outliers) in the box plot are assigned the values of Q1 − 1.5 × IQR and Q3 + 1.5 × IQR, respectively. The mean values are presented in small squares (■) inside the box bodies. Datasets ● of *Malus* taxa are shown on the right of each box plot, and their distributions are fitted with a line. (**e**) The intraspecific uniformity of pollen morphology traits using 15% as the criteria. If $\bar{c.v.}$ ≤ 15%, then the trait has met the uniformity requirements. (**f**) The interspecific distinctness of pollen morphology traits using 15% as the criteria. If *c.v*. ≥ 15%, the differentiation degree of this trait is considered to be high among all the taxa. The one-way ANOVA (Tukey’s method) was performed to obtain a more accurate expression. * indicates significant differences (*p* < 0.05) in pollen morphology traits between *Malus* taxa.

All SEM micrographs of pollen grains of the *Malus* taxa and corresponding data are shown in Figure S1 [21] and Table S1, respectively. The aberrant pollen rates exhibited considerable variation, ranging from 0% to 95.60% (Figure 1a). A total of 13.08% of the taxa had an abnormal pollen rate exceeding 40%. Nevertheless, most taxa (75.70%) displayed abnormal pollen rates below 20.00%. The morphological characteristics of normal pollen grains are summarized below.

The average length of the polar axis (P) of the *Malus* taxa was 44.95 μm (Figure 1b). The smallest mean P was found for pollen of *M. honanensis* (29.44 μm), while the largest mean P was found for ‘Brandywine’ (52.35 μm). The mean equator diameter (E) was 23.65 μm, ranging from 20.95 μm in ‘Van Eseltine’ to 29.64 μm in ‘Pink Princess’. The area of the equatorial view with two colpi (S) ranged from 560.25 to 1108.89 μm^2^. The P/E ratio ranged from 1.22 to 2.21, the P/E′ ratio ranged from 1.60 to 2.56, and the E′/E_0_ ratio ranged from 0.76 to 0.88 (Figure 1c). The ridge width (RW) of the striate exine ornamentation ranged from 0.13 to 0.25 μm, and the furrow width (FW) of the striate ranged from 0.04 to 0.28 μm (Figure 1d). A total of 35 taxa had no perforations (32.71%), while 72 taxa had perforations, with perforation densities (PDs) ranging from 0.11 per μm^2^ in *M. prunifolia* to 11.18 per μm^2^ in ‘Van Eseltine’.

In summary, according to the pollen size classification created by Erdtman [22], most pollen was medium-sized (25.1–50 μm; 89.7%), and a few taxa were relatively large (10.3%) (Figure 1a). All pollen grains were bilaterally symmetrical, with perprolate, prolate, or sub-rectangular shapes in the equatorial view (P/E = 1.22–2.21) and tricolpate with three germinal furrows in the polar view. Exine ornamentation was striate–perforate; some were striate or smooth (‘Regal’) without perforations.

### 2.2. Intraspecifc Uniformity Test and Interspecific Distinctness Analysis of Pollen Morphology Traits

Compared with pollen size and morphology, ornamentation was highly interspecifically differentiated and intraspecifically unstable (Figure 1d,e). Except for PD and FW of the striate, the remaining seven traits had significant intraspecific uniformity ($\bar{c.v.}$ ≤ 15%) (Figure 1d). As for the interspecific distinctness of pollen traits, all traits differed significantly among the taxa (*p* < 0.0001) (Figure 1e). However, pollen extine ornamentation (PD, FW, and RW) showed a higher degree of distinctness (*c.v*. ≥ 15%). Because the PD and FW vary significantly within the taxa, the RW of striate ornamentation was more suitable as the main index for identification.

### 2.3. Cluster Analysis Based on Pollen’s Phenotypic Traits

**Figure 2:**
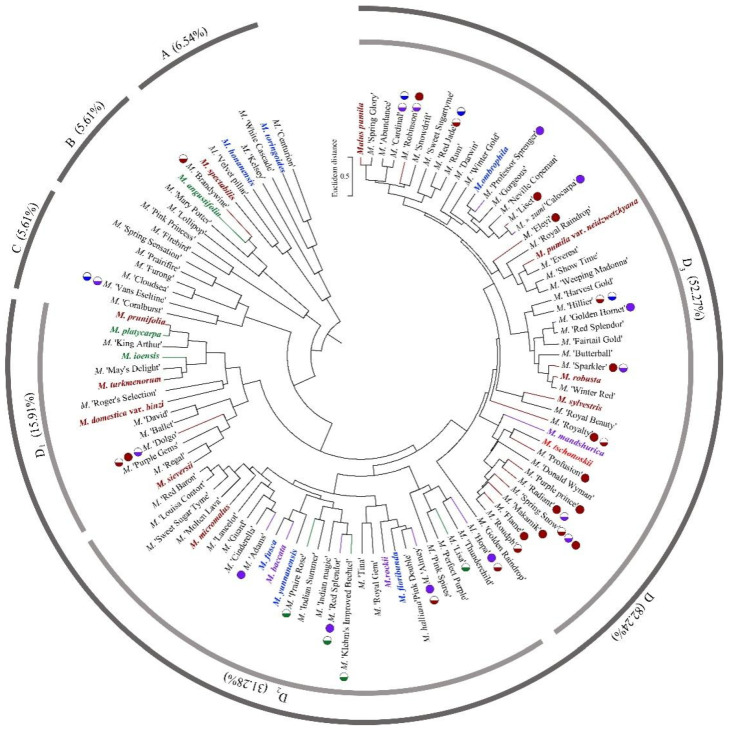
The cluster analysis of the pollen’s phenotypic traits in 107 *Malus* taxa. The scientific names of the species are noted in different colors. The same color font indicates they are in the same section [17]. The red font represents the species belonging to Sect. *Docyniopsis*. The green font represents the species belonging to Sect. *Chloromeles*. The blue font represents the species belonging to Sect. *Sorbomalus*. The purple font represents the species belonging to Sect. *Baccatus*. The brown font represents the species belonging to Sect. *Malus*. A fully filled circle indicates that these cultivars are grouped with their parents, and a half-filled circle indicates a cultivar that is not grouped with its parent. Parental traceability information for each cultivar is available in the study by Zhou et al. [20].

A cluster analysis of *Malus* taxa based on six independent variables (P, E, E′/E, RW, FW, and PD) was conducted (Figure 2). The 107 taxa were divided into four groups: A, B, C, and D. The pollen size (P, E, and S) and morphological parameters (P/E, P/E′, and E′/E) of the A group were significantly smaller than those of the other groups (*p* < 0.0001, Table 1). The pollen size of the B group was significantly greater than that of the other groups (*p* < 0.0001). The PD and morphological parameters (E′/E) of the C group were significantly greater than those of the other groups (*p* < 0.0001). There was no apparent trend for the pollen phenotypic parameters in the D group. The germplasm distribution was uneven among the four groups, with 82.2% of the taxa in the D group. The D group was divided into three subgroups (D_1_, D_2_, and D_3_). The number of taxa in the three D group subgroups was also uneven (D_1_: 15.9%, D_2_: 31.8%, and D_3_: 52.3%), and the pollen size of the D_1_ and D_2_ subgroups was significantly higher than that of the D_3_ subgroup (*p* < 0.0001). The pollen morphology parameters of the D_2_ and D_3_ subgroups were significantly greater than those in the D_1_ subgroup (*p* < 0.0001). The D_2_ subgroup had significantly greater PD than the other two subgroups (*p* < 0.0001).

### 2.4. The Characteristics of Family Aggregation Distribution

#### 2.4.1. The Family Aggregation Distribution between Species and Their Sections

**Figure 3:**
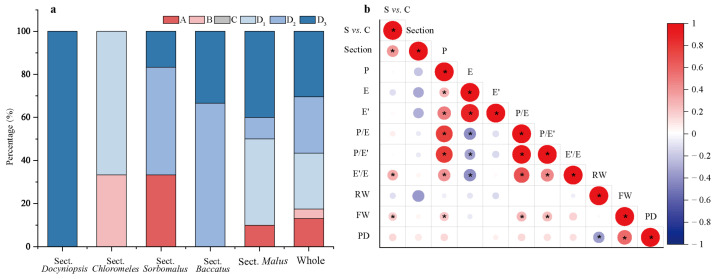
The distribution of *Malus* sections in each cluster group and their Pearson correlation with pollen phenotypic traits. (**a**) The distribution of *Malus* species of *Malus* sections in each cluster group. (**b**) Pearson correlation analysis between *Malus* classification units and pollen phenotypic traits. According to the order from original to evolved in the classic taxonomy system, the five sections of 23 *Malus* species were assigned the following values: Sect. *Docyniopsis* (1) → Sect. *Chloromeles* (2) → Sect. *Sorbomalus* (3) → Sect. *Baccatus* (4) → Sect. *Malus* (5). S vs. C represents species and cultivars populations, valued to species (1) and cultivars (2) in Pearson correlation analysis. The circle marked with ‘*’ indicates that the correlation reached a significant level (*p* < 0.05).

According to the *Malus* taxonomy system proposed by Rehder [16] and Li [17], the genus *Malus* can be classified into six sections (*Docyniopsis*, *Chloromeles*, *Sorbomalus, Baccatus*, *Malus,* and *Eriolobus*). The 23 species used in this study represent all sections except for *Eriolobus*: I, Sect. *Docyniopsis* (1 species); II, Sect. *Chloromeles* (3 species); III, Sect. *Sorbomalus* (6 species); IV, Sect. *Baccatus* (3 species) and Sect. *Malus* (10 species) (Figure 2). The species distribution within the same section was relatively concentrated (Figure 2 and Figure 3a). None of the species we studied were classified in the C group. All species in Sect. *Docyniopsis* and *Baccatus* were distributed in the D group. Two-thirds of the species in Sect. *Chloromeles* and *Sorbomalus* were distributed in the D group, and 90% of species belonging to Sect. *Malus* were distributed in the D group. The total percentage of the family aggregation distribution reached 82.6% in the same group and 52.2% in the same subgroups (Figure 3a).

According to the order from original to evolved in the classic taxonomy system [17], the five sections of *Malus* species were valued as follows: Sect. *Docyniopsis* (1) → Sect. *Chloromeles* (2) → Sect. *Sorbomalus* (3) → Sect. *Baccatus* (4) → Sect. *Malus* (5). There was a negative but insignificant correlation between the five sections and pollen size (r < −0.3, *p* > 0.05) (Figure 3b).

#### 2.4.2. The Family Aggregation Distribution between Cultivars and Their Parents (Species)

In this study, we could trace the origin of the parents of 28 cultivars (Table 2, Figure 2). Cultivars in the A group had no traceable parents, whereas cultivars in the B and C groups each had one traceable parent. There were 26 traceable cultivars in the D group, and all exhibited family aggregation. Overall, according to the statistical analysis of the four cluster groups (A, B, C, and D), the family aggregation distribution of the cultivars was as high as 92.9%. According to our statistical analysis of the six cluster groups/subgroups, the percentage of parent tendency distribution was as high as 67.9%.

Furthermore, E′/E, FW, and PD showed an increasing trend from species to cultivar populations. The positive correlation between populations E′/E and FW reached a significant level (*p* = 0.0015, *p* = 0.039) (Figure 3b). The percentage of the progeny whose perforation density is higher than that of its parents is 53.57%. If the perforation density of the unknown/untested taxa is assumed to be lower, this percentage can be up to 96.43%.

#### 2.4.3. Hybridization Frequency of Species

**Figure 4:**
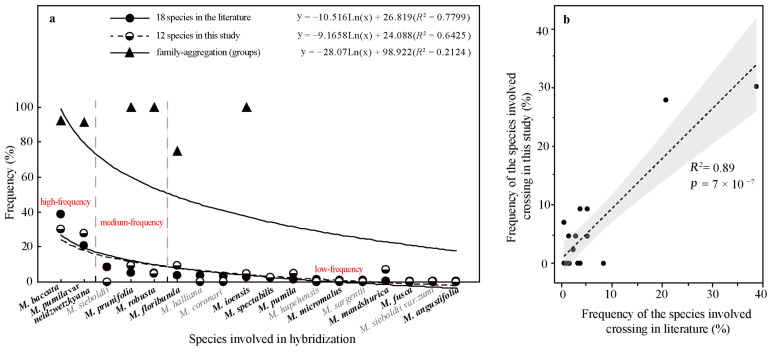
The frequency statistics and correlation analysis of the *Malus* species involved in hybridization. (**a**) The frequency of hybridization and family aggregation of the 18 species based on the literature and this study (12 bolded species). (**b**) The correlation of the hybridization frequency between the species in this study and the literature.

The hybridization route of 65 cultivars is recorded in the literature, involving 18 *Malus* species [17,18,23,24]. The hybridization frequency of the 18 species can be classified into high-, medium-, and low-frequency parents (Figure 4a). *M. baccata* and *M. pumila* var. *neidzwetzkyana* are high-frequency parents, with hybridization frequencies of 38.7% and 20.7%, respectively. We found that our hybridization frequencies for 12 species in this study were highly correlated with what has been recorded in the literature (*R*^2^ = 0.89) (Figure 4b).

## 3. Discussion

### 3.1. High Aberrant Pollen Rates May Be an Effective Indicator for Apomixis or Multiploid Cultivar Selection

The occurrence of aberrant pollen is regular in various angiosperm families [25,26]. These aberrant pollen grains can be distinguished by features such as shape and size, aperture number and arrangement, and ornamentation type, different from the typical pollen morphology of the species. Halbritter et al. [27] pointed out some apomictic *Malus* species that only produce irregularly shaped pollen due to asexual reproduction. In this study, we found that 10.28% of taxa had abnormal pollen proportions exceeding 40%. Of these taxa with high abnormal pollen rates, some, including *M. mandshurica*, ‘Mary Potter’, ‘Regal’, and ‘Firebird’, have been proven to be apomixis and multiploid [28], with a low pollen germination rate [29]. The multiploid plants in the *Malus* genus fail to produce viable pollen during meiosis due to unequal chromosome allocation, which may be the reason for the aberrant pollen [30]. If the apomixis characteristics of *Malus* taxa with aberrant pollen are confirmed, the grafting breeding method for these cultivars can be replaced by seed propagation. Moreover, if confirmed, the high aberrant pollen rate can serve as an effective indicator for apomixis or multiploid cultivar selection.

### 3.2. Variation Tendency of Malus Pollen Morphology

Plants evolve at both macro and micro levels. Intra-genus (inter-species and inter-cultivar) evolution is also considered as microevolution, which reflects the evolutionary process of plant populations within a short term. Inter-genus (or above: inter-family, inter-ordo) evolution can also be regarded as macroevolution, which reflects the origin and the phylogenetic process of plant populations within a geologic age [24]. Different research approaches are required because macroevolution differs from microevolution in terms of the research object type and the time scale. Paleobiology and comparative morphology are often used to investigate macroevolution, whereas genetics, ecology, and systematics are mainly used to investigate microevolution [31].

Current studies have shown that the evolutionary patterns of pollen are observed at the macroevolutionary level. By using both fossil and sample pollen and based on morphological phylogenetics, Walker et al. [13,32,33] studied the evolutionary trend (at macro- and multi-levels) of the pollen morphology of inter-family or inter-generic taxonomic groups and concluded that the evolution directions were as follows: pollen size: large → small; pollen shape: boat-shaped → globose; exine ornamentation: smooth → foveolate and ditch-shaped → clavate, drumstick-shaped and echinate → crisped, reticulum and striate; perforation: nonexistence →existence/small → large; ridge interval: short → long. However, for taxonomic groups of infrageneric germplasms (microlevel), trends in evolution are either highly defined [34,35] or undetectable [4,36].

**Figure 5:**
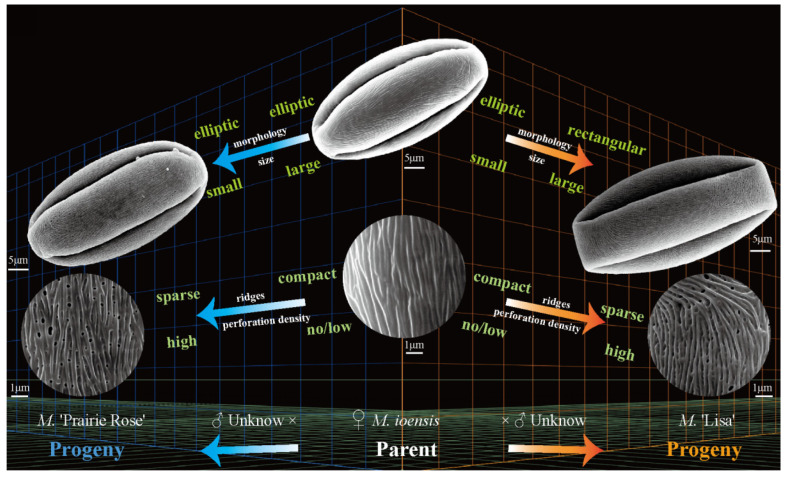
A schematic diagram illustrating the directional variation at the microscopic level of pollen using *M. ioensis* and its naturally pollinated progeny as a case.

The present study investigated nine traits of pollen size, shape, and exine ornamentation of *Malus* taxa. We compared the changes in pollen traits from *Malus* species to cultivars and from the original section to the evolutionary section (microlevel taxonomic groups) via correlation analysis. We determined the following trends: big pollen → small ones (insignificant); elliptic morphologies → rectangular ones (E′/E, significant); large and compact ridges → small and sparse ones (RW, insignificant; FW, significant); no perforations or low perforation densities → high perforation densities (PD, insignificant) (Figure 5). Our conclusion agrees well with the viewpoint of Walker et al. [13,33,34]. Moreover, FW and PD had low intraspecific uniformity ($\bar{c.v.}$ > 15%), which means that the distribution of perforations and stripes on the exine was uneven. RW was more suitable as the main index for identification ($\bar{c.v.}$ < 15%, *c*.*v*. > 15%). E′/E, as the new morphological index, had high intraspecific uniformity and directivity of evolution, which is better for comparing the degree of originality or evolution of *Malus* taxa.

Notably, we observed a higher density of perforations in the D_2_ cluster, which comprised the evolutionary sections, including Sect. *Baccatus* and Sect. *Sorbomalus* and their progeny. These evolutionary groups were characterized by many perforations, such as *M. baccata*, *M. rockii*, *M. floribunda*, and *M. fusca* (Table S1). The number of perforations in their progeny may be influenced by the characteristics of their parental species. Furthermore, we calculated the percentage of the progeny exhibiting an upward trend in perforation density across generations. We discovered that over half (53.57%) of the progeny displayed perforation densities exceeding those of their parents (Table 2). This trend of increasing variation in perforation density, from lower to higher, is unequivocally evident in macroevolution (tectate–imperforate → tectate–perforate → semitectate → intectate) [32,37]. The adaptive significance of this evolutionary trend in perforation was related to the evolution of an infratectal–intercolumellar storage area for sporophytic incompatibility proteins [32]. This protein may be stored within the perforations or reticulum [32]. However, it is unclear if the increased perforation density in hybrid offspring indicates a more pronounced self-incompatibility in subsequent generations. This hypothesis warrants further investigation for validation.

### 3.3. Genetic Relationships of Malus Taxa Based on Phenotypic Traits of Pollen

*Malus* has various germplasm and complex genetic relationships [17]. Many studies have explored the genetic relationships between *Malus* species and cultivars using molecular markers [38,39], isozymes [40], and palynology [41]. However, these studies had small sample sizes and did not analyze the entire genus. Previous studies also did not analyze the genetic relationship of *Malus* using statistical methods.

The classic taxonomic system of *Malus* is based on plant morphological characteristics [16,17]. Here, using the cluster analysis of phenotypic traits in *Malus* pollen, we found that the distribution of *Malus* species from the same section was relatively concentrated within groups. The family aggregation distribution percentage was 92.9%, which indicated that the classification system based on pollen phenotypic traits was consistent with the traditional classification system based on phenotypic traits.

There were 18 species involved in crossing, and *M. baccata* and *M. pumilar* var. *neidzwetzkyana* were determined to be high-frequency parents (38.7% and 20.7%, respectively). *M. baccata* is widely distributed in China and has large white flowers [42]. *M. pumilar* var. *neidzwetzkyana* is the genetic source of red flower cultivars. There is no red color germplasm in the original species except for *M. pumilar* var. *neidzwetzkyana,* which has great significance in color improvement breeding [43]. In this study, the species belong to the D_2_ and D_3_ subgroups, respectively. These two subgroups not only have a higher percentage of *Malus* cultivars but also have a high family aggregation distribution. This is in accordance with Fiala [18] and Jefferson [19]. Meanwhile, this also verified why the phenotypic diversity of existing crabapple cultivars is not rich enough. There are some excellent germplasms, such as *M. ioensis, M. hupehensis, M. halliana,* and *M. micromalus*. If these germplasms can be frequently used as parents to carry out crossbreeding, their offspring will likely be able to fill vacancies in existing cultivars (few early blooms, few orange fruits, and a faint aroma).

Future research could focus on establishing connections between phenotypic variations and taxonomic specificities, functionality, fruit yield, and the phenotypes of flowers, leaves, and fruits.

## 4. Materials and Methods

### 4.1. Materials

In all, 107 *Malus* taxa (including 23 species and 84 cultivars) were used (Table 3). Pollen was collected from the national repository of *Malus* germplasm (lat. 32°42′ N, long. 119°55′ E, Yangzhou City, Jiangsu Province, China). The tree ages were between 5 and 8 years. We collected 30 flowers at the large bud stage, wrapped them in litmus paper, laid them in a cooler at 4 °C, and transported them back to the laboratory within 8 h. Anthers were peeled from the flowers and air dried at room temperature for one week, after which the pollen sacs were split open. The dried pollen was collected for further observation.

### 4.2. Pollen Observations

The dried pollen grains were spread on a glass slide with a double-sided adhesive tape and were sprayed with gold for 120 s at an electric current of 16 mA using a magnetron ion-sputtering device (E-1010, Hitachi Ltd., Tokyo, Japan). Then, a field-emission scanning electron microscope (SEM; S-4800, Hitachi Ltd., Japan) was used to observe pollen traits. The sample support was kept at room temperature, and the acceleration voltage was 15 kV. Representative pollen grains were photographed (2.50 K) and presented in the book [21].

**Figure 6:**
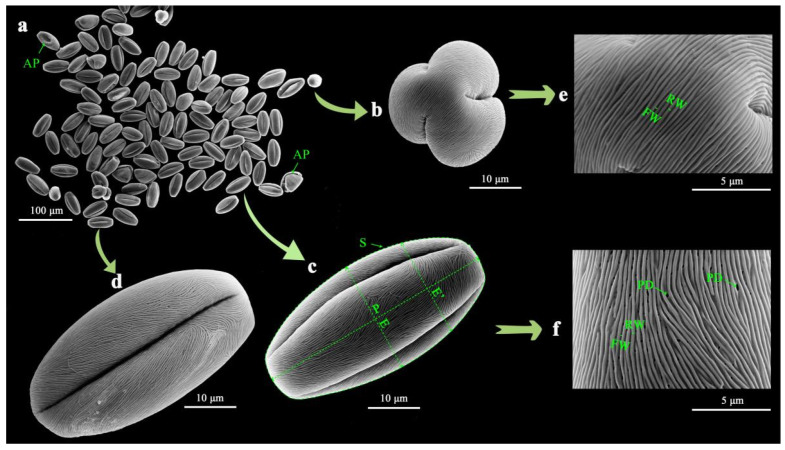
The pollen morphology of *Malus* ‘Amey’ obtained via SEM observation. (**a**) The population; (**b**) The polar view; (**c**) Two colpi in the equatorial view; (**d**) One colpi in the equatorial view; (**e**) Ornamentation in the polar view; (**f**) Ornamentation in the equatorial view. The indicators contain the abnormal pollen rate (AP), the length of the polar axis (P), equatorial diameter (E), the diameter at the equatorial plane halfway between the equator and pole (E′), the ridge width (RW), the furrow width (FW), and the perforation density (PD). The area of the equatorial view with two colpi (S) serves as a relative metric computed by Image J [44] to estimate the region encompassed within the equatorial plane.

Image J 1.54 software [44] was used to measure the abnormal pollen rate (AP), pollen size, morphology, and ornamentation according to original SEM micrographs of pollen grains (Figure 6) [21]. Pollen grains that differ in shape, size, number arrangement of apertures, and ornamentation type from the typical pollen type of the species are considered as abnormal pollen. The percentage of total pollen count that consists of abnormal pollen is defined as the abnormal pollen rate. The pollen size indicators measured were the length of the polar axis (P), equatorial diameter (E), the diameter at the equatorial plane halfway between the equator and pole (E′), and the area of equatorial view with two colpi (S). The pollen morphology indicators measured were the ratio between the length of the polar axis and equatorial diameter (P/E), the ratio of the length of the polar axis and diameter at the equatorial plane halfway between the equator and pole (P/E′), and the ratio between the diameter at the equatorial plane halfway between the equator and the pole and the equatorial diameter (E′/E). The pollen ornamentation indicators measured were the ridge width (RW), furrow width (FW), and perforation density (PD). Thirty replicates were used for each indicator.

### 4.3. Data Analysis

Family aggregation means that the progeny and their parents were gathered via cluster analysis, which can provide a reference for their genetic relationship.

The intraspecific uniformity test for quantitative traits is expressed by the mean coefficient of variation ($\bar{c.v.}$). If $\bar{c.v.}$ ≤ 15%, then the trait has met the uniformity requirements. The interspecific distinctness analysis of quantitative traits was expressed by the coefficient of variation (*c.v*.) of the mean value of each trait in all taxa. If *c.v*. ≥ 15%, the differentiation degree of this trait is high among all the taxa.

(1) $$\bar{c.v.}=\frac{1}{n}\sum_{i=1}^{n}{(S}_{i}/\bar{X_{i}})$$

(2) $$c.v.=S^{\prime }/{\bar{X}}^{\prime }\times 100\%$$

Here, n denotes the number of taxa; *S*_i_ and ${\bar{X}}_{i}$ denote the rank for the standard deviation of the observed values and the mean observed values for each trait in each taxon’s repetitions, respectively. S′ and $\bar{X}$′ denote the standard deviation and the average of observed mean values of each trait in all taxa, respectively.

OriginPro 9.8.0.200 (OriginLab Corp., Northampton, MA, USA) and Adobe Illustrator CC 23.0.2 (Adobe Inc., San Jose, CA, USA) were used for processing data and plotting graphs. ANOVA was conducted using SPSS 26.0 (IBM Corp., Armonk, NY, USA).

## 5. Conclusions

In this study, we explored the characteristics of the phenotypic variation of *Malus* pollen at the infrageneric scale. Overall, (1) an aberrant pollen rate and ornamentation were highly interspecifically differentiated, but ornamentation was also intraspecifically unstable, especially for perforation densities. (2) The pollen alteration direction from the original to the evolutionary population of *Malus* was from large to small, from elliptic to rectangular morphologies, from large and compact to small and sparse ridges, and from low to high perforation densities. (3) The distribution of family aggregation represents a notable characteristic. In summary, this study provides a reference for identifying *Malus*’ pedigree relationships. These findings improve our understanding of pollen phenotypic variation’s taxonomic significance at the genus level.

## Figures and Tables

**Table 1:** The pollen phenotypic traits of *Malus* taxa based on cluster analysis.

Pollen Phenotypic Traits	Group/Subgroup						
	A	B	C	D	D_1_	D_2_	D_3_
P (μm)	36.26 ± 4.21 d	48.42 ± 4.36 a	46.54 ± 2.69 abc	45.29 ± 3.01 bc	46.18 ± 3.58 abc	47.14 ± 2.26 ab	43.90 ± 2.54 c
E (μm)	24.24 ± 1.02 bc	27.42 ± 0.82 a	22.95 ± 1.33 de	23.39 ± 1.11 de	24.86 ± 0.76 b	23.72 ± 0.85 cd	22.74 ± 0.79 e
E′ (μm)	19.25 ± 1.10 c	22.41 ± 0.67 a	19.85 ± 1.20 bc	19.78 ± 0.90 bc	20.49 ± 0.76 b	20.14 ± 0.83 b	19.35 ± 0.75 c
S (μm^2^)	690.05 ± 74.49 e	1 043.46 ± 71.34 a	840.68 ± 77.43 bcd	834.03 ± 83.22 cd	902.58 ± 76.27 b	879.84 ± 64.92 bc	785.28 ± 63.12 d
P/E	1.51 ± 0.21 d	1.78 ± 0.20 c	2.04 ± 0.13 a	1.94 ± 0.11 ab	1.86 ± 0.15 bc	1.99 ± 0.08 a	1.94 ± 0.10 ab
P/E′	1.90 ± 0.24 c	2.17 ± 0.19 b	2.36 ± 0.15 a	2.30 ± 0.13 a	2.27 ± 0.19 ab	2.35 ± 0.09 a	2.28 ± 0.12 ab
E′/E	0.80 ± 0.02 d	0.82 ± 0.03 c	0.87 ± 0.00 a	0.85 ± 0.02 b	0.82 ± 0.01 c	0.85 ± 0.01 b	0.85 ± 0.01 b
RW (μm)	0.19 ± 0.04 a	0.20 ± 0.02 a	0.17 ± 0.03 a	0.19 ± 0.03 a	0.18 ± 0.06 a	0.17 ± 0.02 a	0.20 ± 0.03 a
FW (μm)	0.10 ± 0.05 c	0.12 ± 0.04 bc	0.20 ± 0.04 a	0.12 ± 0.04 bc	0.10 ± 0.05 c	0.15 ± 0.03 b	0.12 ± 0.03 bc
PD (No./μm^2^)	3.66 ± 3.49 b	1.81 ± 1.92 c	9.23 ± 1.41 a	1.87 ± 2.23 c	0.42 ± 0.64 c	4.72 ± 1.53 b	0.57 ± 0.81 c
Taxa quantity (No. and %)	7 and 6.54	6 and 5.61	6 and 5.61	88 and 82.24	14 and 15.91	28 and 31.82	46 and 52.27

The different lowercase letters indicate significant differences (*p* < 0.05) in each pollen morphology trait among different groups or subgroups.

**Table 2:** The parent traceability and identification of family aggregation distribution characteristics of *Malus* cultivars.

No.	Progeny	Breeding Routes	References	Class Group in Figure 2	Is the Family- Aggregation Distributed?		Is the Perforation Density of the Progeny Higher than that of one of Its Parents?	
					A, B, C, D	A, B, C, D_1_, D_2_, D_3_	Unknown/Untested Taxa Are Considered with Lower Perforation Densities.	Unknown/Untested Taxa Are Excluded.
1	*M*. ‘Adams’	*M. baccata* × unknown → *M*. ‘Adams’	[19]	D_2_ × unknown → D_2_	Yes	Yes	Yes	Yes
2	*M*. ‘Almey’	*M. baccata* × *M. pumila* var. *neidzwetzkyana* → *M*. ‘Almey’	[19]	D_2_ × D_3_ → D_2_	Yes	Yes	Yes	Yes
3	*M*. ‘Brandywine’	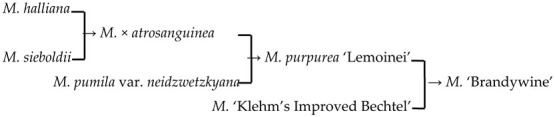	[18,19]	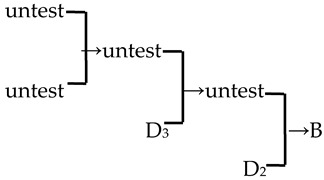	No	No	Yes	No
4	*M*. ‘Cardinal’	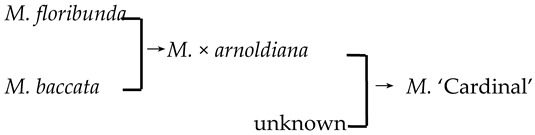	[18,19]	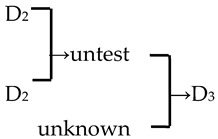	Yes	No	Yes	No
5	*M*. ‘Dolgo’	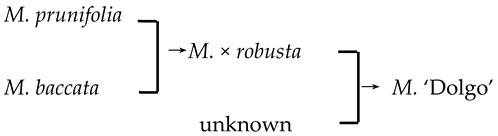	[18]	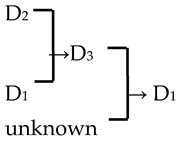	Yes	Yes	Yes	Yes
6	*M*. ‘Eleyi’	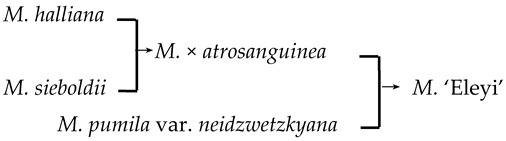	[19]	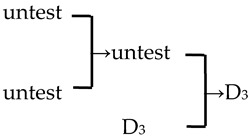	Yes	Yes	Yes	No
7	*M*. ‘Flame’	*M. pumila* × unknown → *M*. ‘Flame’	[18]	D_3_ × unknown → D_3_	Yes	Yes	Yes	No
8	*M*. ‘Golden Hornet’	*M. mandshurica* × *M. sieboldii* → *M*. ‘Golden Hornet’	[19]	D_3_ × untest→ D_3_	Yes	Yes	Yes	Yes
9	*M*. ‘Hillier’	*M. floribunda* × *M. prunifolia* → *M*. ‘Hillier’	[23]	D_2_ × D_1_ → D_3_	Yes	No	Yes	Yes
10	*M*. ‘Hopa’	*M. baccata* × *M. pumila* var. *neidzwetzkyana* → *M*. ‘Hopa’	[18]	D_3_ × D_3_→ D_3_	Yes	Yes	Yes	Yes
11	*M*. ‘Klehm’s Improved Bechtel’	*M. ioensis* × unknown → *M*. ‘Klehm’s Improved Bechtel’	[17]	D_1_ × unknown → D_2_	Yes	No	Yes	Yes
12	*M*. ‘Lisa’	*M. ioensis* × unknown → *M*. ‘Lisa’	[18]	D_1_ × unknown → D_2_	Yes	No	Yes	Yes
13	*M*. ‘Liset’	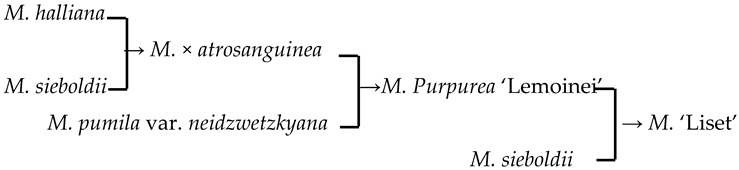	[18]	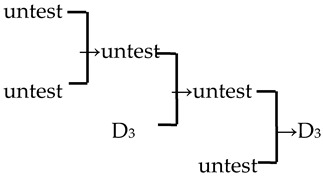	Yes	Yes	Yes	No
14	*M*. ‘Makamik’	*M. pumila* var. *neidzwetzkyana* × Unknown → *M*. ‘Makamik’	[18]	D_3_ × unknown → D_3_	Yes	Yes	Yes	No
15	*M*. ‘Prairie Rose’	*M. ioensis* × unknown → *M*. ‘Prairie Rose’	[18]	D_1_ × unknown → D_2_	Yes	No	Yes	Yes
16	*M*. ‘Professor Sprenger’	*M. mandshurica* × *M. sieboldii* → *M*. ‘Professor Sprenger’	[23]	D_3_ × untest → D_3_	Yes	Yes	Yes	Yes
17	*M*. ‘Profusion’	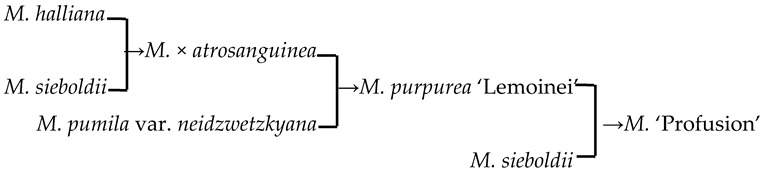	[19]	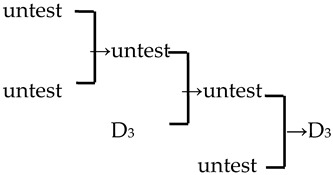	Yes	Yes	Yes	No
18	*M*. ‘Purple prince’	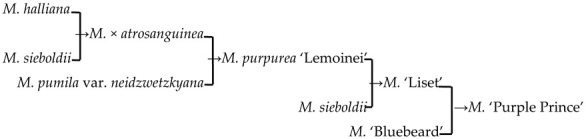	[18]	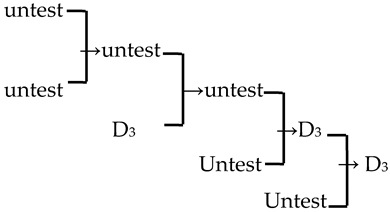	Yes	Yes	Yes	Yes
19	*M*. ‘Radiant’	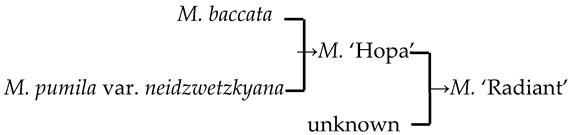	[19]	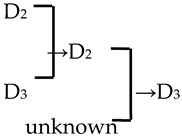	Yes	Yes	Yes	No
20	*M*. ‘Red Jade’	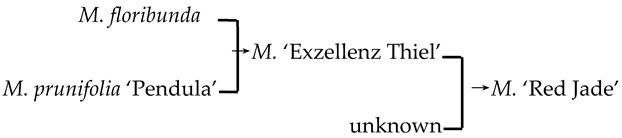	[19]	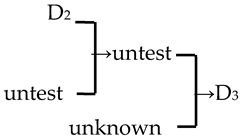	Yes	No	Yes	No
21	*M*. ‘Red Splendor’	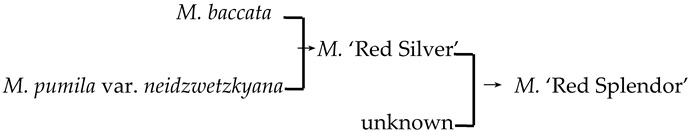	[19]	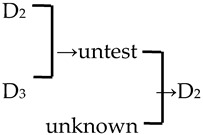	Yes	Yes	Yes	Yes
22	*M*. ‘Robinson’	*M. baccata* × *M. pumila* → *M*. ‘Robinson’	[19]	D_2_ × D_3_ → D_3_	Yes	Yes	No	No
23	*M*. ‘Royalty’	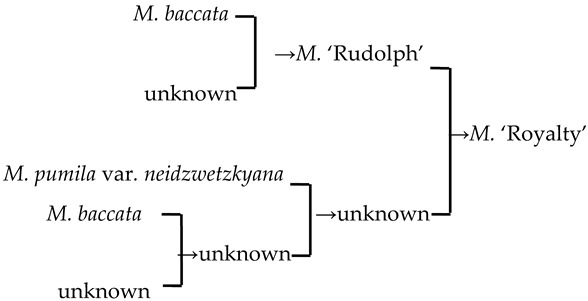	[19]	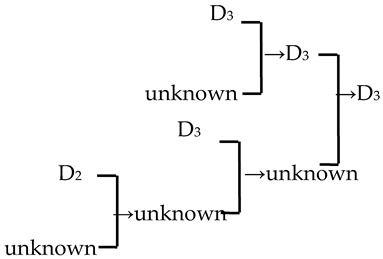	Yes	Yes	Yes	No
24	*M*. ‘Rudolph’	*M. baccata* × unknown→ *M*. ‘Rudolph’	[18,19]	D_2_ × unknown →D_3_	Yes	No	Yes	No
25	*M*. ‘Sparkler’	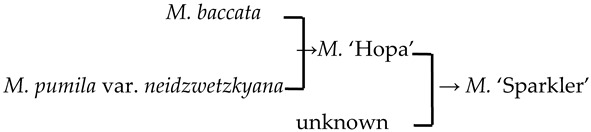	[18]	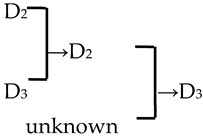	Yes	Yes	Yes	No
26	*M*. ‘Spring Snow’	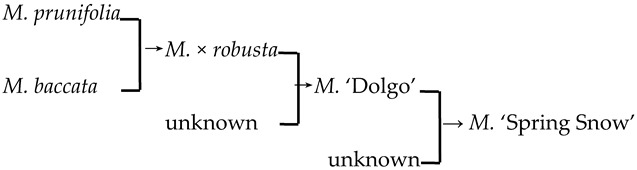	[19]	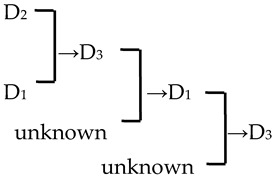	Yes	Yes	Yes	Yes
27	‘Van Eseltine’	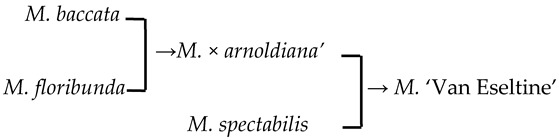	[19]	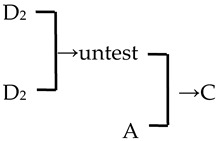	No	No	Yes	Yes
28	*M. zumi* ‘Calocarpa’	*M. mandshurica* × *M. sieboldii* → *M. zumi* ‘Calocarpa’	[17]	D_3_ × untest →D_3_	Yes	Yes	Yes	Yes
The probability of family aggregation distribution (the percentage of the progeny and its parents belonging to the same group, %)					92.86	67.86	/	/
The percentage of the progeny whose perforation density is higher than that of its parents (%)					/	/	96.43	53.57

**Table 3:** A list of the 107 taxa collected from the national repository of the *Malus* taxa.

Species	Cultivars	Cultivars	Cultivars
*Malus angustifolia* ^II^	*M*. ‘Abundance’	***M*. ‘Hopa’**	*M*. ‘Red Sentinel’
*M. baccata* ^IV^	***M*. ‘Adams’**	*M*. ‘Indian Magic’	***M*. ‘Red Splendor’**
*M. domestica* var. *binzi* ^V^	***M*. ‘Almey’**	*M*. ‘Indian Summer’	*M*. ‘Regal’
*M. floribunda* ^V^	*M*. ‘Ballet’	*M*. ‘Kelsey’	***M*. ‘Robinson’**
*M. fusca* ^III^	***M*. ‘Brandywine’**	*M*. ‘King Arthur’	*M*. ‘Roger’s Selection’
*M. honanensis* ^III^	*M*. ‘Butterball’	***M*. ‘Klehm’s Improve Bechtel’**	*M*. ‘Royal Beauty’
*M. ioensis* ^II^	***M*. ‘Cardinal’**	*M*. ‘Lancelot’	*M*. ‘Royal Gem’
*M. mandshurica* ^IV^	*M*. ‘Centurion’	***M*. ‘Lisa’**	*M*. ‘Royal Raindrop’
*M. micromalus* ^V^	*M*. ‘Cinderella’	***M*. ‘Liset’**	***M*. ‘Royalty’**
*M. ombrophila* ^III^	*M*. ‘Cloudsea’	*M*. ‘Lollipop’	***M*. ‘Rudolph’**
*M. platycarpa* ^II^	***M*. ‘Coralburst’**	*M*. ‘Louisa Contort’	*M*. ‘Rum’
*M. prunifolia* ^V^	*M*. ‘Darwin’	***M*. ‘Makamik’**	*M*. ‘Show Time’
*M. pumila* ^V^	*M*. ‘David’	***M*. ‘Mary Potter’**	*M*. ‘Snowdrift
*M. Pumila* var. *neidzwetzkyana* ^V^	***M*. ‘Dolgo’**	*M*. ‘May’s Delight’	***M*. ‘Sparkler’**
*M. robusta* ^V^	*M*. ‘Donald Wyman’	*M*. ‘Molten Lava’	*M*. ‘Spring Glory’
*M. rockii* ^IV^	***M*. ‘Eleyi’**	*M*. ‘Perfect Purple’	*M*. ‘Spring Sensation’
*M. sieversii* ^V^	*M*. ‘Everest’	*M*. ‘Pink Princess’	***M*. ‘Spring Snow’**
*M. spectabilis* ^V^	*M*. ‘Fairytail Gold’	*M*. ‘Pink Spires’	*M*. ‘Sugar Tyme’
*M. sylvestris* ^V^	*M*. ‘Firebird’	***M*. ‘Prairie Rose’**	*M*. ‘Sweet Sugar Tyme’
*M. toringoides* ^III^	***M*. ‘Flame’**	*M*. ‘Prairifire’	*M*. ‘Thunderchild’
*M. tschonoskii* ^I^	*M*. ‘Furong’	***M*. ‘Professor Sprenger’**	***M*. ‘Tina’**
*M. turkmenorum* ^V^	***M*. ‘Golden Hornet’**	***M*. ‘Profusion’**	***M*. ‘Vans Eseltine’**
*M. yunnanensis* ^III^	*M*. ‘Golden Raindrop’	*M*. ‘Purple Gems’	*M*. ‘Velvet Pillar’
	***M*. ‘Gorgeous’**	***M*. ‘Purple Prince’**	*M*. ‘Weeping Madonna’
	*M*. ‘Guard’	*M. purpurei* ‘Neville Copeman’	*M*. ‘White Cascade’
	*M. halliana* ‘Pink Double’	***M*. ‘Radiant’**	***M*. ‘Winter Gold’**
	*M*. ‘Harvest Gold’	*M*. ‘Red Baron’	*M*. ‘Winter Red
	***M*. ‘Hillier’**	***M*. ‘Red Jade’**	***M. zumi* ‘Calocarpa’**

The superscript numbering from I to V indicates the evolutionary sequence of sections and series, with I being the most ancient and V being the most advanced. I: Sect. *Docyniopsis*, II: Sect. *Chloromeles*, III: Sect. *Sorbomalus*, IV: Sect. *Baccatus*, V: Sect. *Malus* [16,17]. Cultivars in bold font indicate that their parentage is known, according to Zhou et al. [20].

## Data Availability

Data are available upon request from the author.

## Supplementary Materials

The following supporting information can be downloaded at: https://www.mdpi.com/article/10.3390/plants13172522/s1, Figure S1:Pollen electron micrographs of *Malus* taxa.; Table S1: The pollen phenotypic traits of 107 *Malus* taxa.

## References

[1] Blackmore S., Wortley A.H., Skvarla J.J., Rowley J.R. (2007). Pollen Wall Development in Flowering Plants. New Phytol..

[2] Katifori E., Alben S., Cerda E., Nelson D.R., Dumais J. (2010). Foldable Structures and the Natural Design of Pollen Grains. Proc. Natl. Acad. Sci. USA.

[3] Sarwar A.K.M.G., Takahashi H. (2012). Pollen Morphology of *Kalmia* L. (Phyllodoceae, Ericaceae) and its Taxonomic Significance. Bangladesh J. Plant Taxon..

[4] Lechowicz K., Wrońska-Pilarek D., Bocianowski J., Maliński T. (2020). Pollen Morphology of Polish Species from the Genus *Rubus* L. (Rosaceae) and its Systematic Importance. PLoS ONE.

[5] Jardine P.E., Palazzesi L., Tellería M.C., Barreda V.D. (2022). Why does Pollen Morphology Vary? Evolutionary Dynamics and Morphospace Occupation in the Largest Angiosperm Order (Asterales). New Phytol..

[6] Nazeri J.V. (2008). Pollen Morphology of the Genus *Malus* (Rosaceae). Iran. J. Sci. Technol. (Sci.).

[7] Li M., Tian C.-F., Idrees M., Pathak M., Xiong X.-H., Gao X.-F., Wang X.-R. (2023). Pollen Morphology in *Sorbus* L. (Rosaceae) and Its Taxonomic Implications. Plants.

[8] Romeiro L.d.A., da Silva E.F., Vasconcelos L.V., Lopes K.d.S., Carreira L.M.M., Guimarães J.T.F. (2023). Pollen Morphology of Convolvulaceae from Southeastern Amazonian Cangas and Its Relevance for Interaction Networks and Paleoenvironmental Studies. Plants.

[9] Banks H., Forest F., Lewis G. (2014). Evolution and Diversity of Pollen Morphology in Tribe Cercideae (Leguminosae). Taxon.

[10] Tellería M.C., Barreda V.D., Jardine P.E., Palazzesi L. (2023). The use of Pollen Morphology to Disentangle the Origin, Early Evolution, and Diversification of the Asteraceae. Int. J. Plant Sci..

[11] Gonçalves-Esteves V., Vieira G.R.M., Carvalho R.J.P.D., Crespo S.R.D.M., Mendonça C.B.F. (2020). Pollen Morphology of some Species of *Spermacoceae* s.s. (Rubiaceae) of the Atlantic Forest, Rio de Janeiro, Brazil. Acta Bot. Bras..

[12] Sarwar A.K.M.G., Hoshino Y., Araki H. (2015). Pollen Morphology and Its Taxonomic Significance in the Genus *Bomarea* Mirb. (Alstroemeriaceae)—I. Subgenera *Baccata*, *Sphaerine*, and *Wichuraea*. Acta Bot. Bras..

[13] Walker J.W., Walker A.G. (1984). Ultrastructure of Lower Cretaceous Angiosperm Pollen and the Origin and Early Evolution of Flowering Plants. Ann. Mo. Bot. Gard..

[14] Quamar M.F., Singh P., Garg A., Tripathi S., Farooqui A., Shukla A.N., Prasad N. (2022). Pollen Characters And Their Evolutionary and Taxonomic Significance: Using Light and Confocal Laser Scanning Microscope to Study Diverse Plant Pollen Taxa from Central India. Palynology.

[15] Guo L., Zhou S.L., Zhang Z.S., Shen X., Cao Y., Zhang D.L., Shu H.R. (2009). Relationships of Species, Hybrid Species and Cultivars in Genus *Malus* Revealed by AFLP Markers. Sci. Silvae Sin..

[16] Rehder A. (1940). Manual of Cultivated Trees and Shrubs.

[17] Li Y.N. (2001). Researches of Germplasm Resources of Malus Mill.

[18] Fiala J.L. (1994). Flowering Crabapples: The genus Malus.

[19] Jefferson R.M. (1970). History, Progeny, and Locations of Crabapples of Documented Authentic Origin.

[20] Zhou T., Shen X., Zhou D.J., Fan J.J., Zhao M.M., Zhang W.X., Cao F.L. (2018). Advances in the Classification of Crabapple Cultivars. Acta Hortic. Sin..

[21] Zhang W.X., Fan J.J., Xie Y.F., Peng Y., Zhou T., Zhao M.M. (2018). An Illustrated Electron Microscopic Study of Crabapple Pollen.

[22] Erdtman G. (1969). Handbook of Palynology: An Introduction to the Study of Polllen Grains and Spores.

[23] Zheng Y., Qu X., Guo L., Sun F.Y., Mao Z.Q., Shen X. (2008). Advances on Ornamental Crabapple Resources. J. Shandong Agric. Univ. (Nat. Sci. Ed.).

[24] Reznick D.N., Ricklefs R.E. (2009). Darwin’s Bridge between Microevolution and Macroevolution. Nature.

[25] Pozhidaev A.E., Harley M.M., Morton C.M., Blackmore S. (2000). Pollen Variety and Aperture Patterning. Pollen and Spores: Morphology and Biology.

[26] Banks H., Stafford P., Crane P.R. (2007). Aperture variation in the pollen of *Nelumbo* (Nelumbonaceae). Grana.

[27] Halbritter H., Ulrich S., Grímsson F., Weber M., Zetter R., Hesse M., Buchner R., Svojtka M., Frosch-Radivo A. (2018). Illustrated Pollen Terminology.

[28] Olien W.C. (1987). Apomictic Crabapples and Their Potential for Research and Fruit Production. HortScience.

[29] Wei Z.Q., Peng Y., Li F.X., Di C.Y., Zhang W.X. (2023). Study on the Cross Compatibility and Parent Selection among Ornamental Crabapple Cultivars. Acta Bot. Boreal.-Occident. Sin..

[30] Mao B.Q., Chin M.H., Liu C.J., Zheng B., Fan M.H. (1995). A Research on the Ability of Apomixis in *Malus* Plants. J. Southwest Univ..

[31] Xu B.S. (1991). An Overview of Macroevolution on the Viewpoint of Microevolution. Acta Bot. Yunnanica.

[32] Walker J.W. (1974). Evolution of Exine Structure in the Pollen of Primitive Angiosperms. Am. J. Bot..

[33] Walker J.W., Walker A.G. (1980). Comparative Pollen Morphology of the Mainland African Genera of Myristicaceae (Cephalosphaera, Coelocaryon, Pycnanthus, and Scyphocephalium). Am. J. Bot..

[34] Welsh M., Stefanović S., Costea M. (2010). Pollen Evolution and its Taxonomic Significance in *Cuscuta*, (dodders, Convolvulaceae). Plant Syst. Evol..

[35] Akhila H., Beevy S.S. (2015). Palynological Characterization of Species of *Sesamum* (Pedaliaceae) from Kerala: A Systematic Approach. Plant Syst. Evol..

[36] Yang X.H. (1986). Observation and study on *Malus* pollen. J. Southwest Agric. Univ..

[37] Lee S., Heo K.I., Cho J., Lee C., Chen W., Kim S.C. (2011). New Insights into Pollen Morphology and Its Implications in the Phylogeny of *Sanguisorba* L. (Rosaceae; Sanguisorbeae). Plant Syst. Evol..

[38] Gasi F., Simon S., Pojskic N., Kurtovic M., Pejic I. (2010). Genetic Assessment of Apple Germplasm in Bosnia and Herzegovina Using Microsatellite and Morphologic Markers. Sci. Horti..

[39] Patzak J., Paprštein F., Henychová A., Sedlák J. (2012). Comparison of Genetic Diversity Structure Analyses of SSR Molecular Marker Data within Apple (*Malus* × domestica) Genetic Resources. Genome.

[40] Wagner I., Weeden N.F. (2000). Isozymes in *Malus sylvestris*, *Malus domestica* and in related *Malus* species. Acta Hortic..

[41] Zhang W.X., Zhao M.M., Fan J.J., Zhou T., Chen Y.X., Cao F.L. (2017). Study on Relationship between Pollen Exine Ornamentation Pattern and Germplasm Evolution in Flowering Crabapple. Sci. Rep..

[42] Chen X., Li S., Zhang D., Han M., Xin J., Zhao C., Wang S., Xin L., Ma J., Ji J. (2019). Sequencing of a Wild Apple (*Malus baccata*) Genome Unravels the Differences between Cultivated and Wild Apple Species Regarding Disease Resistance and Cold Tolerance. G3 Genes Genomes Genet..

[43] Yang M., Che S., Zhang Y., Song W., Yan G., Yu W. (2019). *Malus niedzwetzkyana* (Dieck) Langenf Transcriptome Comparison and Phylogenetic Analysis with *Malus sieversii* (Ledeb) Roem. Genet. Resour. Crop Evol..

[44] Schneider C.A., Rasband W.S., Eliceiri K.W. (2012). Nih Image to Imagej: 25 Years of Image Analysis. Nat. Methods.

