# Supplementary material for: Characteristics of Phenotypic Variation of Malus Pollen at Infrageneric Scale
Source: Plants (Basel). 2024 Sep 8;13(17):2522. doi: 10.3390/plants13172522 (PMC11397718; doi:10.3390/plants13172522)
Supplement: Supplementary file 1 [file plants-13-02522-s001.zip › Figure S1.pdf]

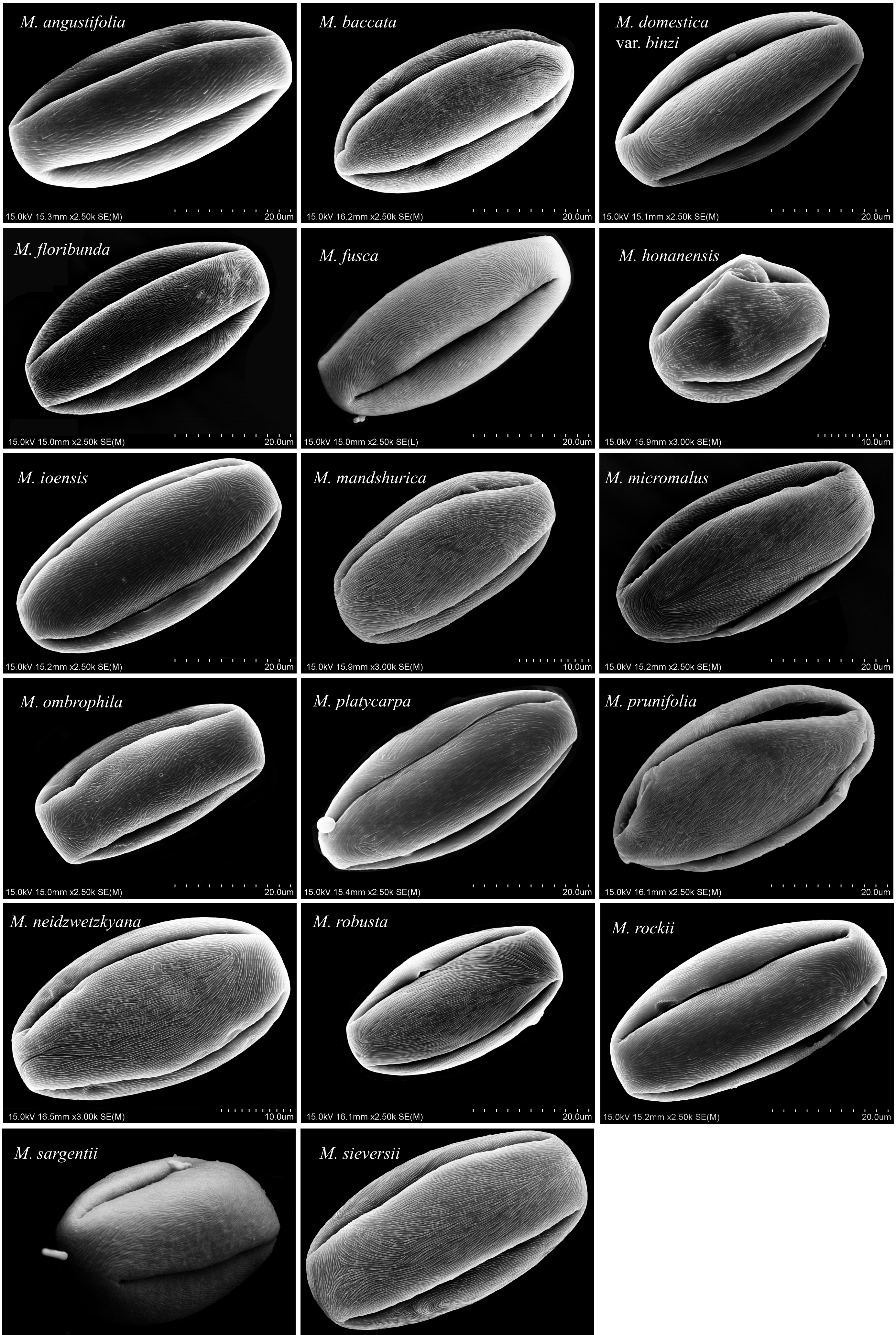
M. angustifolia
15.0kV 15.3mm x2.50k SE(M)
20.0um
M. baccata
15.0kV 16.2mm x2.50k SE(M)
20.0um
M. domestica var. binzi
15.0kV 15.1mm x2.50k SE(M)
20.0um
M. floribunda
15.0kV 15.0mm x2.50k SE(M)
20.0um
M. fusca
15.0kV 15.0mm x2.50k SE(L)
20.0um
M. honanensis
15.0kV 15.9mm x3.00k SE(M)
10.0um
M. ioensis
15.0kV 15.2mm x2.50k SE(M)
20.0um
M. mandshurica
15.0kV 15.9mm x3.00k SE(M)
10.0um
M. micromalus
15.0kV 15.2mm x2.50k SE(M)
20.0um
M. ombrophila
15.0kV 15.0mm x2.50k SE(M)
20.0um
M. platycarpa
15.0kV 15.4mm x2.50k SE(M)
20.0um
M. prunifolia
15.0kV 16.1mm x2.50k SE(M)
20.0um
M. neidzwetzkyana
15.0kV 16.5mm x3.00k SE(M)
10.0um
M. robusta
15.0kV 16.1mm x2.50k SE(M)
20.0um
M. rockii
15.0kV 15.2mm x2.50k SE(M)
20.0um
M. sargentii
15.0kV 14.5mm x3.00k SE(L)
10.0um
M. sieversii
15.0kV 16.3mm x3.00k SE(M)
10.0um

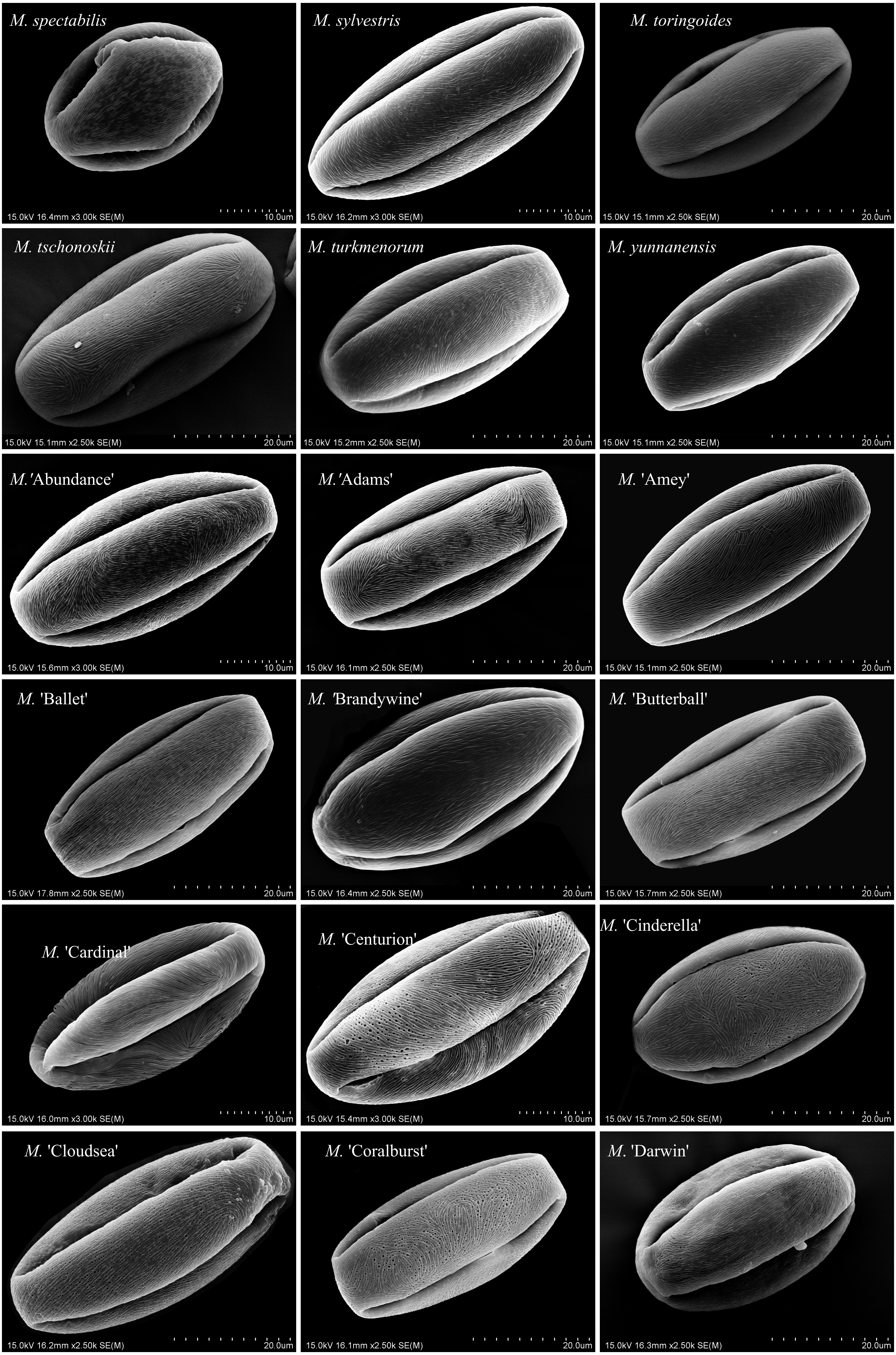
M. spectabilis
15.0kV 16.4mm x3.00k SE(M)
10.0um
M. sylvestris
15.0kV 16.2mm x3.00k SE(M)
10.0um
M. toringoides
15.0kV 15.1mm x2.50k SE(M)
20.0um
M. tschonoskii
15.0kV 15.1mm x2.50k SE(M)
20.0um
M. turkmenorum
15.0kV 15.2mm x2.50k SE(M)
20.0um
M. yunnanensis
15.0kV 15.1mm x2.50k SE(M)
20.0um
M.'Abundance'
15.0kV 15.6mm x3.00k SE(M)
10.0um
M.'Adams'
15.0kV 16.1mm x2.50k SE(M)
20.0um
M. 'Amey'
15.0kV 15.1mm x2.50k SE(M)
20.0um
M. 'Ballet'
15.0kV 17.8mm x2.50k SE(M)
20.0um
M. 'Brandywine'
15.0kV 16.4mm x2.50k SE(M)
20.0um
M. 'Butterball'
15.0kV 15.7mm x2.50k SE(M)
20.0um
M. 'Cardinal'
15.0kV 16.0mm x3.00k SE(M)
10.0um
M. 'Centurion'
15.0kV 15.4mm x3.00k SE(M)
10.0um
M. 'Cinderella'
15.0kV 15.7mm x2.50k SE(M)
20.0um
M. 'Cloudsea'
15.0kV 16.2mm x2.50k SE(M)
20.0um
M. 'Coralburst'
15.0kV 16.1mm x2.50k SE(M)
20.0um
M. 'Darwin'
15.0kV 16.3mm x2.50k SE(M)
20.0um

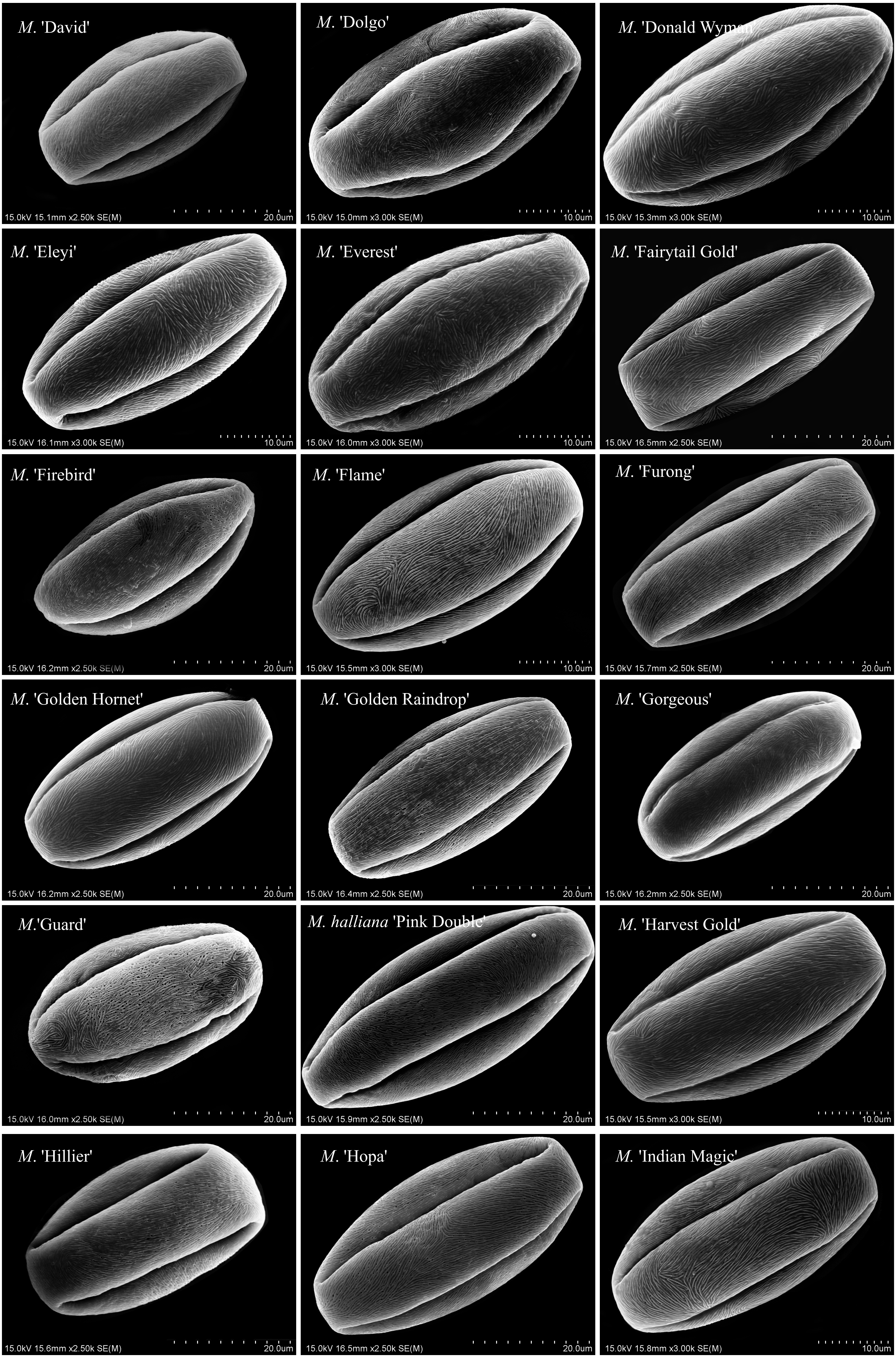
M. 'David'
15.0kV 15.1mm x2.50k SE(M)
20.0um
M. 'Dolgo'
15.0kV 15.0mm x3.00k SE(M)
10.0um
M. 'Donald Wyman'
15.0kV 15.3mm x3.00k SE(M)
10.0um
M. 'Eleyi'
15.0kV 16.1mm x3.00k SE(M)
10.0um
M. 'Everest'
15.0kV 16.0mm x3.00k SE(M)
10.0um
M. 'Fairytail Gold'
15.0kV 16.5mm x2.50k SE(M)
20.0um
M. 'Firebird'
15.0kV 16.2mm x2.50k SE(M)
20.0um
M. 'Flame'
15.0kV 15.5mm x3.00k SE(M)
10.0um
M. 'Furong'
15.0kV 15.7mm x2.50k SE(M)
20.0um
M. 'Golden Hornet'
15.0kV 16.2mm x2.50k SE(M)
20.0um
M. 'Golden Raindrop'
15.0kV 16.4mm x2.50k SE(M)
20.0um
M. 'Gorgeous'
15.0kV 16.2mm x2.50k SE(M)
20.0um
M.'Guard'
15.0kV 16.0mm x2.50k SE(M)
20.0um
M. halliana 'Pink Double'
15.0kV 15.9mm x2.50k SE(M)
20.0um
M. 'Harvest Gold'
15.0kV 15.5mm x3.00k SE(M)
10.0um
M. 'Hillier'
15.0kV 15.6mm x2.50k SE(M)
20.0um
M. 'Hopa'
15.0kV 16.5mm x2.50k SE(M)
20.0um
M. 'Indian Magic'
15.0kV 15.8mm x3.00k SE(M)
10.0um

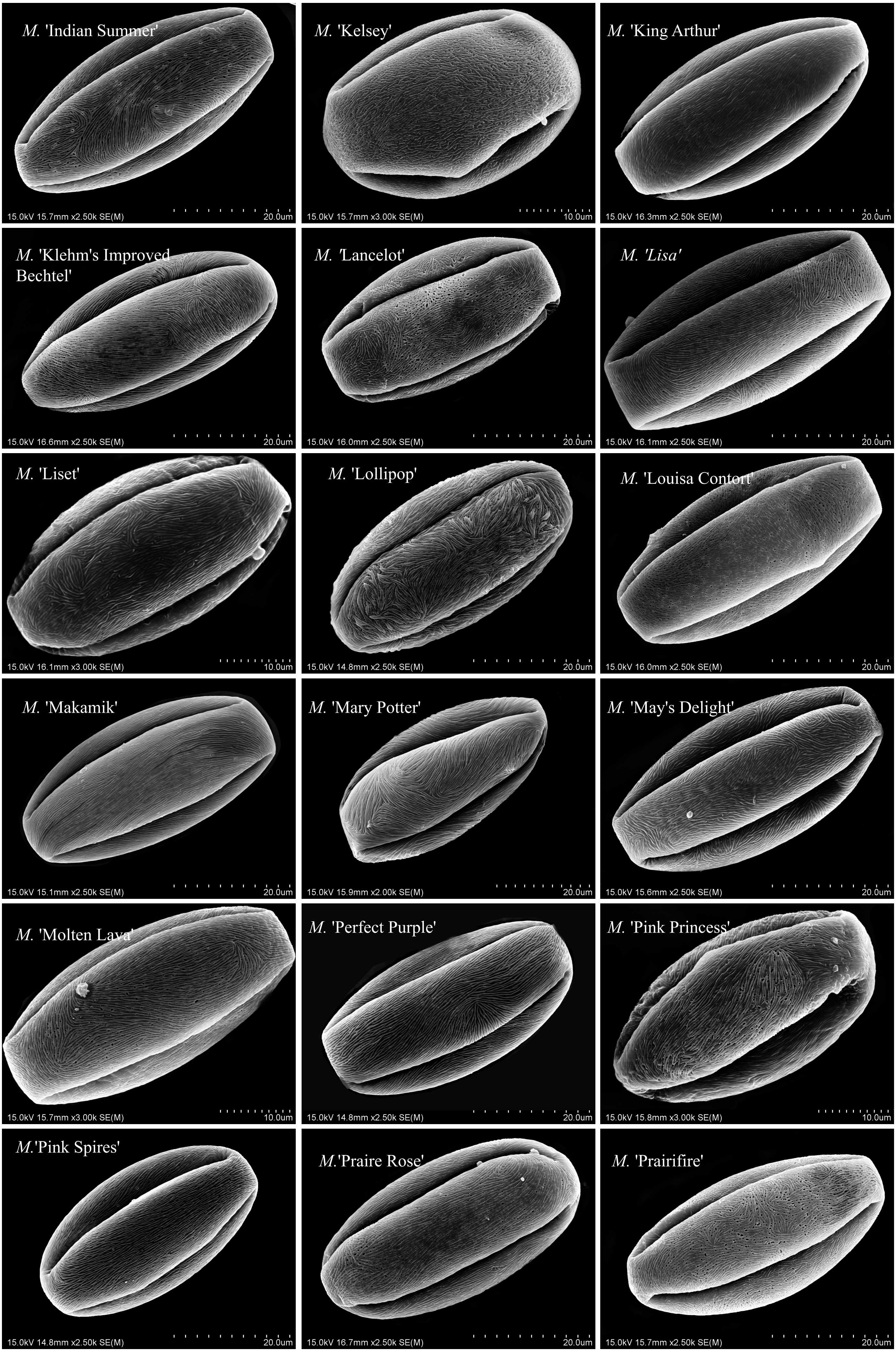
M. 'Indian Summer'
15.0kV 15.7mm x2.50k SE(M)
20.0um
M. 'Kelsey'
15.0kV 15.7mm x3.00k SE(M)
10.0um
M. 'King Arthur'
15.0kV 16.3mm x2.50k SE(M)
20.0um
M. 'Klehm's Improved Bechtel'
15.0kV 16.6mm x2.50k SE(M)
20.0um
M. 'Lancelot'
15.0kV 16.0mm x2.50k SE(M)
20.0um
M. 'Lisa'
15.0kV 16.1mm x2.50k SE(M)
20.0um
M. 'Liset'
15.0kV 16.1mm x3.00k SE(M)
10.0um
M. 'Lollipop'
15.0kV 14.8mm x2.50k SE(M)
20.0um
M. 'Louisa Contort'
15.0kV 16.0mm x2.50k SE(M)
20.0um
M. 'Makamik'
15.0kV 15.1mm x2.50k SE(M)
20.0um
M. 'Mary Potter'
15.0kV 15.9mm x2.00k SE(M)
20.0um
M. 'May's Delight'
15.0kV 15.6mm x2.50k SE(M)
20.0um
M. 'Molten Lava'
15.0kV 15.7mm x3.00k SE(M)
10.0um
M. 'Perfect Purple'
15.0kV 14.8mm x2.50k SE(M)
20.0um
M. 'Pink Princess'
15.0kV 15.8mm x3.00k SE(M)
10.0um
M.'Pink Spires'
15.0kV 14.8mm x2.50k SE(M)
20.0um
M.'Praire Rose'
15.0kV 16.7mm x2.50k SE(M)
20.0um
M. 'Prairifire'
15.0kV 15.7mm x2.50k SE(M)
20.0um

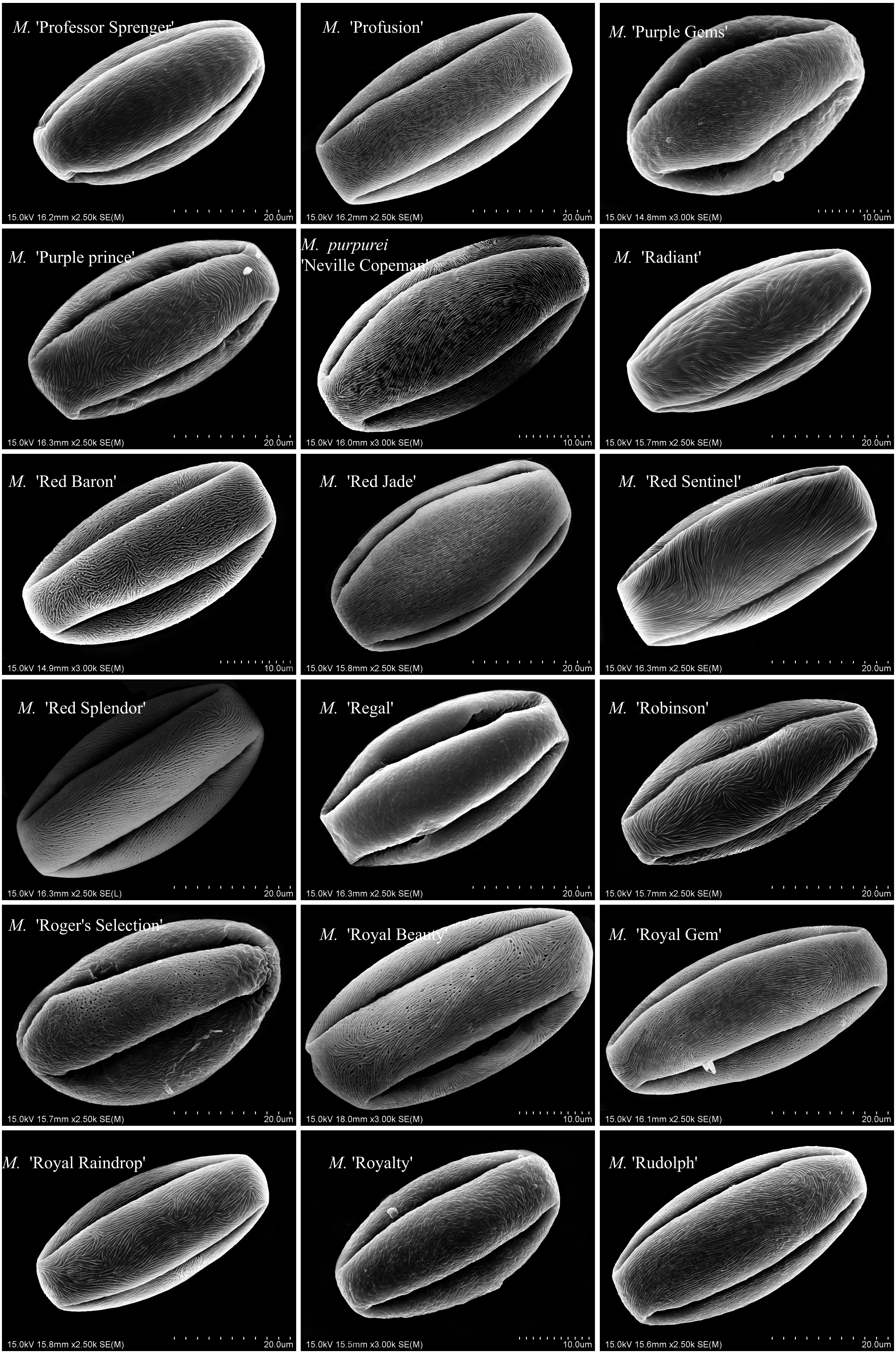
M. 'Professor Sprenger'
15.0kV 16.2mm x2.50k SE(M) 20.0um
M. 'Profusion'
15.0kV 16.2mm x2.50k SE(M) 20.0um
M. 'Purple Gems'
15.0kV 14.8mm x3.00k SE(M) 10.0um
M. 'Purple prince'
15.0kV 16.3mm x2.50k SE(M) 20.0um
M. purpurei 'Neville Copeman'
15.0kV 16.0mm x3.00k SE(M) 10.0um
M. 'Radiant'
15.0kV 15.7mm x2.50k SE(M) 20.0um
M. 'Red Baron'
15.0kV 14.9mm x3.00k SE(M) 10.0um
M. 'Red Jade'
15.0kV 15.8mm x2.50k SE(M) 20.0um
M. 'Red Sentinel'
15.0kV 16.3mm x2.50k SE(M) 20.0um
M. 'Red Splendor'
15.0kV 16.3mm x2.50k SE(L) 20.0um
M. 'Regal'
15.0kV 16.3mm x2.50k SE(M) 20.0um
M. 'Robinson'
15.0kV 15.7mm x2.50k SE(M) 20.0um
M. 'Roger's Selection'
15.0kV 15.7mm x2.50k SE(M) 20.0um
M. 'Royal Beauty'
15.0kV 18.0mm x3.00k SE(M) 10.0um
M. 'Royal Gem'
15.0kV 16.1mm x2.50k SE(M) 20.0um
M. 'Royal Raindrop'
15.0kV 15.8mm x2.50k SE(M) 20.0um
M. 'Royalty'
15.0kV 15.5mm x3.00k SE(M) 10.0um
M. 'Rudolph'
15.0kV 15.6mm x2.50k SE(M) 20.0um

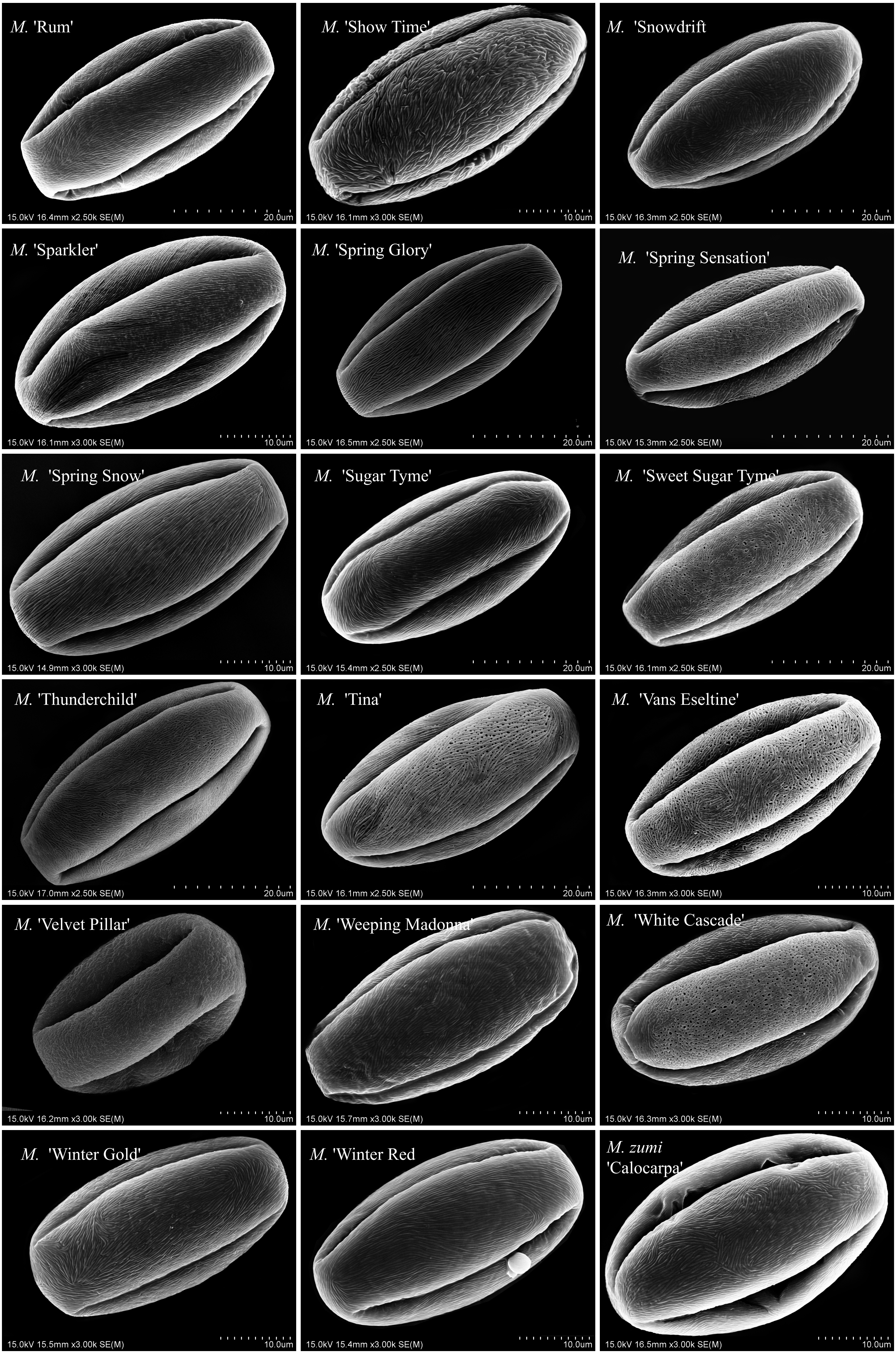

Figure S1 Pollen electron micrographs of *Malus* taxa. These micrographs have been rearranged based on Zhang et al.[21].
