# Supplementary material for: Characteristics of Phenotypic Variation of Malus Pollen at Infrageneric Scale
Source: Plants (Basel). 2024 Sep 8;13(17):2522. doi: 10.3390/plants13172522 (PMC11397718; doi:10.3390/plants13172522)
Supplement: Supplementary file 1 [file plants-13-02522-s001.zip › Table S1.pdf]

Table S1 The phenotypic traits of pollen in 107 *Malus* taxa.

Taxa name	AP (%)	P (μm)	E (μm)	S (μm^2)	P/E	P/E'	E'/E	RW (μm)	FW (μm)	PD (No./μm^2)
M. angustifolia	1.50	52.11±3.12	26.57±1.82	1059.92±12.06	1.96±0.10	2.40±0.14	0.82±0.04	0.22±0.03	0.09±0.03	0.00±0.00
M. baccata	2.22	46.38±1.28	23.43±0.98	853.48±7.12	1.98±0.06	2.36±0.12	0.84±0.03	0.18±0.02	0.15±0.04	2.22±1.82
M. domestica var. binzi	9.23	50.99±3.22	25.13±1.56	868.24±10.22	2.03±0.12	2.55±0.19	0.80±0.04	0.23±0.02	0.13±0.04	0.00±0.00
M. floribunda	10.00	48.02±1.18	24.32±1.34	1100.49±6.22	1.98±0.12	2.34±0.14	0.85±0.03	0.15±0.01	0.13±0.05	2.97±2.22
M. fusca	0	46.87±1.59	23.65±0.93	917.22±3.78	1.98±0.08	2.40±0.09	0.83±0.03	0.16±0.01	0.15±0.06	2.74±2.22
M. honanensis	89.00	29.44±2.66	24.23±1.53	560.25±11.77	1.22±0.12	1.60±0.11	0.76±0.06	0.25±0.04	0.05±0.02	0.39±0.48
M. ioensis	0	51.06±1.80	24.68±1.04	989.72±5.12	2.07±0.10	2.46±0.15	0.84±0.03	0.23±0.03	0.10±0.04	0.00±0.00
M. mandshurica	45.70	45.05±2.51	24.63±1.92	871.46±11.49	1.84±0.14	2.14±0.14	0.86±0.05	0.20±0.02	0.07±0.03	0.00±0.02
M. micromalus	42.30	43.80±2.18	24.56±2.04	844.87±11.04	1.79±0.14	2.13±0.17	0.84±0.05	0.16±0.01	0.10±0.04	4.12±2.30
M. ombrophila	1.25	42.79±2.05	22.61±1.21	691.29±7.98	1.90±0.12	2.23±0.15	0.85±0.06	0.23±0.02	0.15±0.05	0.00±0.00
M. platycarpa	15.83	48.53±1.86	24.38±1.43	929.25±8.33	1.99±0.11	2.44±0.14	0.82±0.04	0.20±0.03	0.05±0.02	0.00±0.00
M. prunifolia	17.80	48.60±1.98	24.22±1.75	924.49±7.79	2.02±0.18	2.47±0.20	0.82±0.03	0.18±0.04	0.09±0.02	0.11±0.26
M. pumila	0.00	45.54±1.33	22.48±1.00	804.04±5.19	2.03±0.10	2.40±0.12	0.84±0.03	0.18±0.02	0.18±0.05	1.25±0.86
M. Pumila varneidzwetzkyana	9.81	41.60±1.50	21.83±1.15	713.24±7.07	1.91±0.11	2.28±0.14	0.84±0.04	0.17±0.03	0.10±0.03	0.76±0.71
M. robusta	7.71	43.89±1.48	22.56±1.40	777.67±8.44	1.95±0.10	2.26±0.11	0.86±0.03	0.19±0.02	0.08±0.02	0.31±0.45
M. rockii	12.05	48.93±2.33	24.56±1.49	943.83±8.26	2.00±0.14	2.35±0.15	0.85±0.04	0.15±0.01	0.11±0.03	5.23±3.11
M. sieversii	5.51	41.97±2.27	24.58±1.44	810.23±9.59	1.71±0.10	2.05±0.14	0.84±0.03	0.17±0.01	0.12±0.05	1.10±1.07
M. spectabilis	8.40	35.32±2.46	24.34±1.73	675.2±10.13	1.46±0.14	1.91±0.17	0.77±0.06	0.16±0.02	0.04±0.01	0.00±0.00
M. sylvestris	5.00	43.98±1.13	21.61±1.11	746.45±5.39	2.04±0.11	2.43±0.15	0.84±0.04	0.17±0.03	0.07±0.02	2.60±2.50
M. toringoides	7.22	39.21±2.44	23.42±1.12	721.23±8.49	1.68±0.12	2.10±0.12	0.80±0.04	0.19±0.02	0.14±0.04	3.65±1.14
M. tschonoskii	0	48.30±2.39	24.17±1.05	916.88±7.16	2.00±0.11	2.34±0.12	0.85±0.03	0.21±0.02	0.12±0.06	0.48±0.79
M. turkmenorum	14.00	49.06±2.59	25.13±1.72	968.3±9.92	1.96±0.13	2.34±0.18	0.84±0.03	0.23±0.02	0.14±0.05	0.00±0.00
M. yunnanensis	4.44	43.83±2.68	23.48±1.40	808.27±9.38	1.87±0.14	2.25±0.19	0.83±0.05	0.21±0.02	0.17±0.05	2.78±1.66
M. 'Abundance'	0	44.95±1.69	22.32±1.30	787.98±8.8	2.02±0.08	2.36±0.13	0.85±0.04	0.17±0.02	0.09±0.03	0.89±0.96
M. 'Adams'	3.97	44.64±1.44	22.87±1.28	801.83±6.47	1.96±0.12	2.30±0.15	0.85±0.03	0.15±0.01	0.16±0.04	4.87±4.01
M. 'Almey'	0	48.82±1.59	24.48±0.81	938.64±4.63	2.00±0.08	2.37±0.12	0.84±0.03	0.18±0.02	0.12±0.03	2.39±1.55
M. 'Ballet'	16.80	42.45±1.67	24.07±1.65	802.5±8.49	1.77±0.12	2.20±0.15	0.81±0.05	0.15±0.02	0.11±0.04	0.49±0.51
M. 'Brandywine'	0.67	52.35±1.93	26.97±1.54	1108.89±7.62	1.95±0.11	2.33±0.14	0.84±0.04	0.21±0.02	0.09±0.04	0.00±0.00
M. 'Butterball'	0	43.20±2.17	22.24±1.58	754.58±11.63	1.95±0.09	2.27±0.11	0.86±0.03	0.17±0.01	0.16±0.05	1.54±2.57
M. 'Cardinal'	82.56	44.66±3.24	22.59±1.68	792.36±14.15	1.98±0.13	2.35±0.20	0.85±0.04	0.16±0.01	0.06±0.02	0.66±0.65
M. 'Centurion'	7.00	41.76±1.59	22.99±1.70	754.03±7.46	1.83±0.16	2.24±0.19	0.82±0.05	0.17±0.02	0.15±0.05	6.40±0.82
M. 'Cinderella'	6.92	44.57±1.64	23.00±1.35	805.12±7.8	1.94±0.11	2.31±0.17	0.84±0.04	0.18±0.02	0.20±0.06	6.72±4.96
M. 'Cloudsea'	31.30	48.84±2.36	22.57±1.88	865.76±11.74	2.17±0.15	2.50±0.17	0.87±0.03	0.13±0.01	0.25±0.10	8.09±3.01
M. 'Coralburst'	20.00	44.54±1.57	22.60±1.09	790.58±6.18	1.97±0.10	2.30±0.14	0.86±0.03	0.16±0.01	0.23±0.07	9.67±1.90
M. 'Darwin'	4.10	40.41±1.68	22.91±1.18	727.12±6.71	1.77±0.11	2.09±0.16	0.85±0.04	0.16±0.02	0.12±0.02	1.83±1.67
M. 'David'	21.3	41.00±3.16	24.08±1.38	775.41±11.54	1.70±0.12	2.09±0.15	0.82±0.04	0.21±0.03	0.18±0.06	1.35±0.69
M. 'Dolgo'	27.50	45.63±2.15	26.26±1.81	941.1±9.48	1.74±0.12	2.08±0.14	0.84±0.05	0.17±0.02	0.08±0.02	0.39±0.58
M. 'Donald Wyman'	17.14	47.70±1.34	23.38±1.06	875.9±5.51	2.04±0.09	2.44±0.12	0.84±0.04	0.22±0.03	0.09±0.03	0.00±0.00
M. 'Eleyi'	2.86	43.16±1.62	22.25±1.35	754.23±8.17	1.94±0.11	2.33±0.14	0.83±0.04	0.21±0.03	0.11±0.03	0.00±0.00
M. 'Everest'	14.00	41.77±1.89	21.83±1.29	716.16±8.6	1.92±0.12	2.30±0.13	0.84±0.04	0.22±0.03	0.14±0.03	0.00±0.00
M. 'Fairytail Gold'	9.14	42.50±1.49	21.98±1.00	733.68±6.51	1.94±0.09	2.24±0.11	0.87±0.03	0.23±0.02	0.11±0.03	0.00±0.00
M. 'Firebird'	83.84	43.90±4.69	27.75±2.70	956.79±20.84	1.58±0.13	2.04±0.21	0.78±0.06	0.18±0.02	0.12±0.04	1.25±1.44
M. 'Flame'	8.90	47.21±2.15	23.41±1.62	868.01±9.9	2.02±0.13	2.36±0.17	0.86±0.04	0.18±0.02	0.15±0.03	0.68±0.62
M. 'Furong'	17.50	50.30±2.25	22.84±1.17	902.31±8.11	2.21±0.11	2.55±0.17	0.87±0.04	0.15±0.01	0.20±0.06	7.18±1.39
M. 'Golden Hornet'	0	43.83±1.57	22.22±1.01	764.9±5.29	1.98±0.12	2.28±0.16	0.87±0.05	0.25±0.02	0.11±0.02	0.00±0.00
M. 'Golden Raindrop'	5.17	44.95±1.62	21.35±0.94	753.73±6.17	2.11±0.10	2.43±0.14	0.87±0.03	0.23±0.04	0.11±0.04	2.51±1.67
M. 'Gorgeous'	35.71	41.89±1.73	22.62±0.95	744.2±6.17	1.85±0.10	2.21±0.12	0.84±0.04	0.21±0.02	0.07±0.02	0.00±0.00
M. 'Guard'	9.29	44.06±2.28	22.03±1.36	762.34±9.6	2.00±0.11	2.38±0.14	0.84±0.04	0.17±0.01	0.19±0.06	5.96±4.36
M. halliana 'Pink Double'	22.50	49.32±1.65	24.06±1.55	931.98±7.35	2.06±0.14	2.46±0.17	0.84±0.05	0.14±0.01	0.11±0.03	4.23±1.79
M. 'Harvest Gold'	15.38	41.39±2.02	22.56±1.46	733.37±8.32	1.84±0.14	2.11±0.13	0.87±0.05	0.19±0.02	0.11±0.04	0.00±0.00
M. 'Hillier'	33.50	40.81±1.89	22.73±1.40	728.54±9.85	1.80±0.09	2.10±0.15	0.86±0.04	0.17±0.02	0.15±0.04	0.55±0.62

Taxa name	AP (%)	P (μm)	E (μm)	S (μm²)	P/E	P/E'	E'/E	RW (μm)	FW (μm)	PD (No./μm²)
M. 'Hopa'	14.17	48.91±2.29	23.93±1.21	919.24±7.89	2.05±0.11	2.37±0.14	0.87±0.03	0.19±0.02	0.17±0.02	5.23±1.51
M. 'Indian Magic'	1.82	45.45±1.75	23.13±1.03	825.66±6.87	1.97±0.09	2.31±0.12	0.85±0.03	0.16±0.01	0.13±0.04	3.04±1.27
M. 'Indian Summer'	0	48.63±1.19	23.17±1.09	884.95±5.17	2.10±0.10	2.48±0.14	0.85±0.04	0.16±0.02	0.14±0.03	4.69±1.72
M. 'Kelsey'	16.84	35.43±2.12	26.17±2.41	728.22±11.76	1.36±0.14	1.65±0.17	0.83±0.06	0.15±0.02	0.13±0.04	4.82±1.05
M. 'King Arthur'	21.22	46.34±1.50	23.85±1.83	868.03±9.38	1.95±0.13	2.38±0.17	0.82±0.04	0.19±0.02	0.07±0.01	0.00±0.00
M. 'Klehm's Improve Bechtel'	5.00	44.93±3.06	22.61±1.50	797.86±12.21	1.99±0.11	2.33±0.15	0.86±0.04	0.17±0.01	0.11±0.03	4.48±2.14
M. 'Lancelot'	18.00	45.20±2.27	23.73±1.10	842.41±7.45	1.91±0.11	2.27±0.14	0.84±0.04	0.15±0.01	0.17±0.05	7.72±3.83
M. 'Lisa'	18.60	50.59±1.53	24.70±1.03	981.41±6.1	2.05±0.07	2.33±0.09	0.88±0.03	0.16±0.02	0.14±0.04	2.48±1.43
M. 'Liset'	9.80	41.82±1.98	23.29±1.30	764.97±7.45	1.80±0.12	2.14±0.15	0.84±0.04	0.24±0.03	0.13±0.03	0.00±0.00
M. 'Lollipop'	69.23	49.92±2.93	26.66±2.52	1045.26±13.58	1.88±0.15	2.25±0.23	0.84±0.05	0.20±0.02	0.10±0.03	4.90±3.11
M. 'Louisa Contort'	9.29	46.19±1.66	23.71±0.92	860.14±5.37	1.95±0.09	2.29±0.14	0.85±0.03	0.17±0.03	0.15±0.03	5.60±2.20
M. 'Makamik'	6.12	48.48±1.31	23.64±1.24	900.12±6.37	2.06±0.10	2.39±0.15	0.86±0.04	0.20±0.02	0.08±0.02	0.36±0.33
M. 'Mary Potter'	41.80	50.15±2.16	27.94±1.92	1100.49±8.82	1.80±0.13	2.14±0.18	0.84±0.05	0.19±0.02	0.14±0.06	1.48±2.20
M. 'May's Delight'	7.58	49.51±1.22	24.98±0.84	971.35±3.92	1.98±0.08	2.39±0.15	0.83±0.05	0.19±0.02	0.13±0.03	0.00±0.00
M. 'Molten Lava'	5.00	46.47±1.39	23.22±1.29	847.47±7.39	2.00±0.09	2.34±0.12	0.86±0.03	0.15±0.01	0.14±0.04	5.61±1.39
M. 'Perfect Purple'	7.59	50.27±1.55	25.51±1.11	1007.18±6.27	1.97±0.07	2.26±0.10	0.87±0.03	0.20±0.01	0.15±0.03	2.69±1.32
M. 'Pink Princess'	4.58	42.09±3.19	28.64±3.00	946.76±14.62	1.48±0.16	1.88±0.20	0.79±0.05	0.18±0.01	0.20±0.05	3.22±1.63
M. 'Pink Spires'	0	51.60±1.42	24.80±0.94	1005.06±5.31	2.08±0.07	2.42±0.12	0.86±0.02	0.16±0.01	0.14±0.04	6.08±2.31
M. 'Praire Rose'	2.00	48.61±1.33	22.53±1.02	860.15±5.08	2.16±0.11	2.56±0.15	0.84±0.03	0.19±0.02	0.18±0.05	3.52±2.66
M. 'Prairifire'	7.00	45.57±1.33	23.91±1.19	855.75±6.44	1.91±0.09	2.22±0.14	0.86±0.04	0.18±0.02	0.21±0.09	9.31±2.24
M. 'Professor Sprenger'	0.77	41.71±1.65	22.99±1.27	753.13±7.23	1.82±0.11	2.15±0.16	0.85±0.04	0.20±0.03	0.14±0.03	0.00±0.00
M. 'Profusion'	16.8	50.56±1.88	24.21±1.01	961.37±5.57	2.09±0.11	2.42±0.11	0.86±0.03	0.16±0.01	0.14±0.04	0.47±0.47
M. 'Purple Gems'	39.60	41.67±2.21	25.86±2.88	846.33±13.06	1.63±0.18	1.97±0.22	0.83±0.05	0.18±0.02	0.05±0.02	0.00±0.00
M. 'Purple Prince'	4.71	46.13±1.77	23.21±1.10	840.91±6.51	1.99±0.10	2.33±0.14	0.86±0.04	0.23±0.02	0.15±0.04	0.00±0.00
M. purpurei 'Neville Copeman'	15.70	42.69±1.86	23.46±1.37	786.58±6.58	1.83±0.13	2.16±0.14	0.84±0.04	0.18±0.02	0.13±0.04	0.92±1.15
M. 'Radiant'	1.42	46.39±0.74	23.06±1.18	840.18±5.15	2.02±0.10	2.37±0.12	0.85±0.04	0.19±0.03	0.08±0.02	0.00±0.00
M. 'Red Baron'	25.90	45.89±5.18	23.56±3.57	849.15±26.47	1.96±0.14	2.31±0.20	0.85±0.05	0.16±0.01	0.15±0.04	5.04±2.11
M. 'Red Jade'	7.41	44.30±1.53	23.49±1.13	817.29±6.43	1.89±0.09	2.22±0.12	0.85±0.04	0.15±0.01	0.12±0.04	0.63±0.61
M. 'Red Sentinel'	3.33	44.03±1.40	22.10±1.17	764.24±6.54	2.00±0.10	2.30±0.13	0.87±0.03	0.24±0.02	0.10±0.04	0.00±0.00
M. 'Red Splendor'	5.38	46.01±1.34	22.24±1.24	803.67±6.28	2.07±0.12	2.41±0.10	0.86±0.03	0.20±0.02	0.16±0.05	3.56±1.81
M. 'Regal'	40.60	43.10±2.15	24.84±1.45	840.85±5.58	1.74±0.15	2.08±0.21	0.84±0.04	0.00±0.00	0.00±0.00	0.00±0.00
M. 'Robinson'	14.80	45.10±2.10	22.22±0.99	787.06±6.03	2.03±0.13	2.43±0.19	0.84±0.05	0.21±0.02	0.15±0.06	0.00±0.00
M. 'Roger's Selection'	37.86	46.63±2.08	26.00±1.38	952.2±7.36	1.80±0.11	2.23±0.17	0.81±0.06	0.19±0.02	0.19±0.04	1.88±2.24
M. 'Royal Beauty'	7.14	41.43±2.24	21.79±1.42	709.03±8.75	1.91±0.15	2.30±0.17	0.83±0.04	0.17±0.02	0.13±0.04	3.15±1.00
M. 'Royal Gem'	28.57	49.02±1.77	24.17±0.99	930.55±5.45	2.03±0.10	2.43±0.17	0.84±0.04	0.16±0.02	0.14±0.03	7.47±1.89
M. 'Royal Raindrop'	0	43.41±1.14	21.35±1.14	727.91±6.68	2.04±0.10	2.40±0.11	0.85±0.04	0.18±0.01	0.19±0.06	1.01±1.05
M. 'Royalty'	81.20	37.75±2.91	22.32±2.41	661.76±17.47	1.70±0.13	2.03±0.18	0.84±0.05	0.18±0.03	0.06±0.02	0.00±0.00
M. 'Rudolph'	12.00	47.51±1.39	23.04±0.90	859.72±4.93	2.06±0.09	2.39±0.14	0.86±0.04	0.24±0.03	0.10±0.02	1.40±1.31
M. 'Rum'	49.66	44.06±1.55	23.04±1.48	797.29±7.47	1.92±0.12	2.25±0.13	0.85±0.04	0.20±0.03	0.11±0.03	0.00±0.00
M. 'Show Time'	8.24	42.04±1.70	21.86±1.01	721.78±6.45	1.93±0.10	2.26±0.16	0.85±0.04	0.25±0.03	0.21±0.09	0.00±0.00
M. 'Snowdrift'	2.31	45.49±1.58	23.03±1.09	822.81±5.83	1.98±0.11	2.37±0.15	0.84±0.03	0.22±0.04	0.14±0.03	0.00±0.00
M. 'Sparkler'	3.85	42.88±2.00	22.44±1.10	755.73±7.83	1.91±0.10	2.21±0.10	0.86±0.03	0.17±0.03	0.11±0.04	0.33±0.24
M. 'Spring Glory'	5.71	45.84±1.88	22.30±0.97	802.86±7.27	2.06±0.08	2.46±0.14	0.84±0.05	0.22±0.02	0.12±0.03	1.74±1.11
M. 'Spring Sensation'	48.70	46.89±1.94	24.86±0.96	915.53±5.48	1.89±0.10	2.18±0.18	0.87±0.05	0.22±0.05	0.18±0.05	9.92±2.25
M. 'Spring Snow'	2.11	46.75±1.65	23.92±0.89	878.28±5.85	1.96±0.07	2.27±0.08	0.86±0.04	0.20±0.02	0.09±0.02	1.00±0.62
M. 'Sugar Tyme'	0	44.76±2.33	23.86±1.22	838.78±6.95	1.88±0.14	2.23±0.14	0.84±0.04	0.20±0.02	0.09±0.04	0.00±0.00
M. 'Sweet Sugar Tyme'	8.57	45.31±1.72	24.28±1.55	864.04±8.50	1.87±0.11	2.22±0.15	0.85±0.04	0.19±0.02	0.14±0.03	5.24±1.64
M. 'Thunderchild'	15.60	50.23±1.26	23.71±0.89	935.37±4.26	2.12±0.09	2.45±0.10	0.87±0.04	0.16±0.02	0.14±0.05	3.23±1.65
M. 'Tina'	16.90	47.24±2.07	24.69±1.27	916.05±6.91	1.92±0.12	2.31±0.18	0.83±0.04	0.17±0.02	0.28±0.08	6.56±1.32
M. 'Vans Eseltine'	10.10	43.12±2.15	20.95±1.04	709.50±8.80	2.06±0.09	2.40±0.16	0.86±0.03	0.17±0.01	0.14±0.04	11.18±2.47
M. 'Velvet Pillar'	95.60	33.23±2.02	23.88±1.77	623.24±11.93	1.40±0.10	1.74±0.10	0.80±0.05	0.22±0.04	0.05±0.01	0.43±0.42
M. 'Weeping Madonna'	0	41.69±1.04	21.54±0.76	705.29±4.34	1.94±0.07	2.29±0.12	0.85±0.04	0.18±0.02	0.11±0.05	0.00±0.00

Taxa name	AP (%)	P (μm)	E (μm)	S (μm^2)	P/E	P/E'	E'/E	RW (μm)	FW (μm)	PD (No./μm^2)
M. 'White Cascade'	86.60	39.44±2.43	24.67±1.42	764.18±10.22	1.60±0.10	2.04±0.16	0.79±0.04	0.18±0.02	0.17±0.06	9.14±3.42
M. 'Winter Gold'	21.50	40.07±1.88	23.59±1.76	742.40±9.22	1.71±0.14	2.01±0.20	0.85±0.04	0.17±0.02	0.09±0.02	0.00±0.00
M. 'Winter Red	0	43.64±1.58	22.61±1.04	774.95±6.54	1.93±0.09	2.24±0.13	0.86±0.04	0.22±0.02	0.07±0.02	0.00±0.00
M. × *zumi* 'Calocarpa'	3.33	41.34±1.96	23.34±1.44	757.81±8.33	1.78±0.12	2.11±0.14	0.84±0.04	0.20±0.02	0.10±0.03	0.00±0.00

Note: Abnormal pollen rate (AP), length of the polar axis (P), equatorial diameter (E), diameter at the equatorial plane halfway between the equator and pole (E'), ridge width (RW), furrow width (FW), perforation density (P, and the area of the equatorial view with two colpi (S).
